# A novel method for determining sex in late term gestational mice based on the external genitalia

**DOI:** 10.1371/journal.pone.0194767

**Published:** 2018-04-04

**Authors:** Laura B. Murdaugh, Haley N. Mendoza-Romero, Eric W. Fish, Scott E. Parnell

**Affiliations:** 1 Bowles Center for Alcohol Studies, University of North Carolina, Chapel Hill, North Carolina, United States of America; 2 Department of Cell Biology and Physiology, University of North Carolina, Chapel Hill, North Carolina, United States of America; 3 Carolina Institute for Developmental Disabilities, University of North Carolina, Chapel Hill, North Carolina, United States of America; Colorado State University, UNITED STATES

## Abstract

In many experiments using fetal mice, it is necessary to determine the sex of the individual fetus. However, other than genotyping for sex-specific genes, there is no convenient, reliable method of sexing mice between gestational day (GD) 16.5 and GD 18.0. We designed a rapid, relatively simple visual method to determine the sex of mouse fetuses in the GD 16.5—GD 18.0 range that can be performed as part of a routine morphological assessment. By examining the genitalia for the presence or absence of key features, raters with minimal experience with the method were able to correctly identify the sex of embryos with 99% accuracy, while raters with no experience were 95% accurate. The critical genital features include: the presence or absence of urethral seam or proximal urethral meatus; the shape of the genitalia, and the presence or absence of an area related to the urethral plate. By comparing these morphological features of the external genitalia, we show a simple, accurate, and fast way to determine the sex of late stage mouse fetuses. Integrating this method into regular morphological assessments will facilitate the determination of sex differences in fetuses between GD 16.5 and GD 18.0.

## Introduction

While the developmental processes underlying murine gonadal differentiation have been studied in detail, a visual method based on the appearance of the external genitalia for sexing of late gestation fetuses has yet to be proposed. A variety of experiments study gestational day (GD–i.e. days post-coitum) 16.5–18.0 mouse fetuses to determine the effects of prenatal manipulation or specific genes [1, 2] on overall embryonic development, or on specific structures [3]. Moreover, previous studies have shown that in both animals [4,5] and humans there are sex specific effects of teratogens and genetic mutations [6,7]. This, combined with the recent NIH directive [8] to make the examination of both males and females and the evaluation of potential sex differences a paramount goal of ongoing research projects means that a simple technique to rapidly determine sex in late gestation fetuses would be a useful tool. We propose, herein, a visual method of sex determination that only uses the physical characteristics of the fetal external genitalia.

Multiple methods currently exist for determining the sex of postnatal rodents based on external features, such as nipple presence, perineal pigmentation, and anogenital distance [9,10]. However, these methods are not appropriate for mouse fetuses in the GD 16.5 to GD 18.0 range as they rely on structures that have yet to develop or are not distinguishable until the postnatal period [11,12]. During this time period sex can be determined by examining the internal reproductive structures, but this requires minor fetal surgery and organ movement to examine the structures around the bladder [11]. Sex can also be reliably determined through genotyping for sex-specific genes, such as *Sry*, but these methods are time-consuming and necessitate significant monetary investment into other resources [11, 13]. Thus, a reliable method to determine the sex of a late gestation embryo based solely on easily visible characteristics would be faster and more economical, than genotyping. Another advantage of a visual method is that it could be used to sex preserved fetuses (e.g. in paraformaldehyde, Bouin’s, or ethanol) generated from previous studies to perform a *post hoc* analysis of sex differences. Thus, the current work sought to develop a method to rapidly determine sex in late gestational age fetuses with high accuracy and reproducibility.

### Overview of genital development

**Fig 1:**
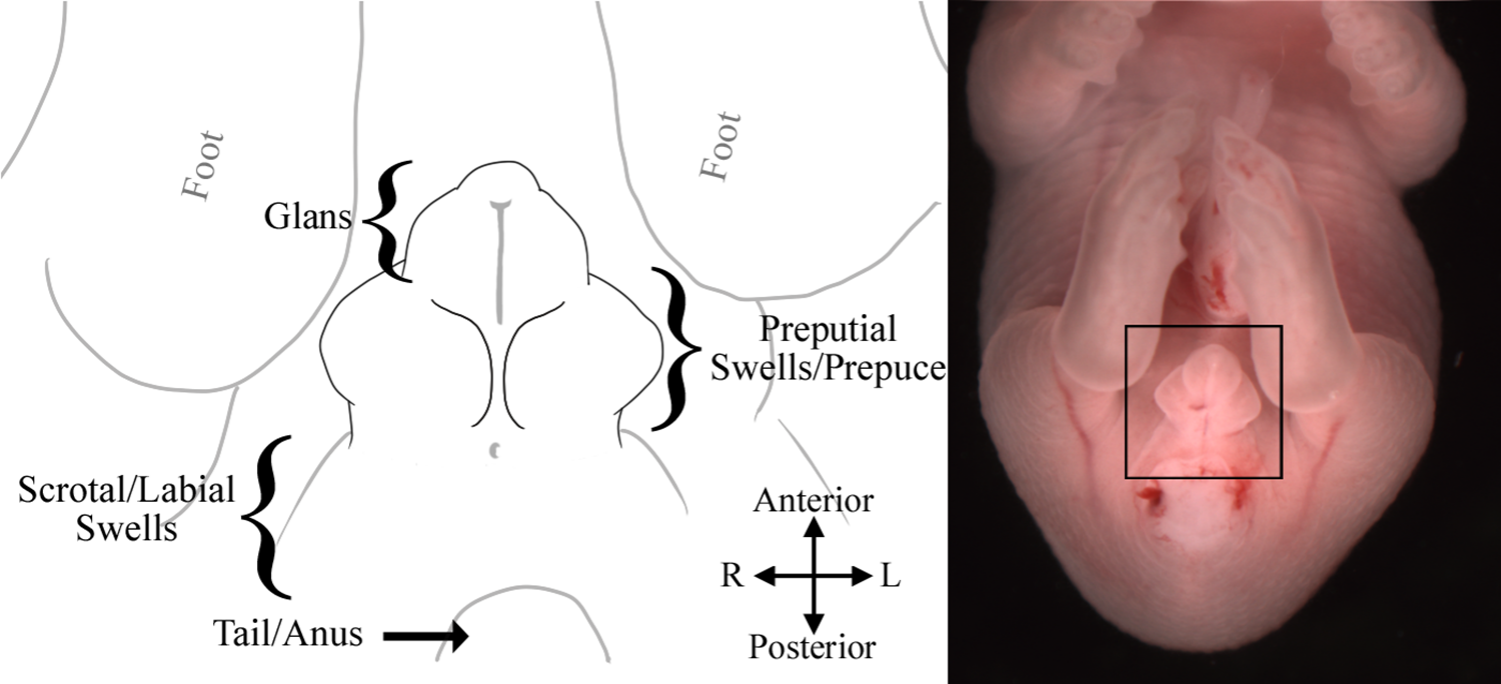
Illustration of mouse external genitalia regions at ~GD 16.5. *Left*, a labeled illustration of the mouse external genitalia as they would appear at ~ GD 16.5. *Right*, a GD 16.5 male with box indicating the area illustrated.

**Fig 2:**
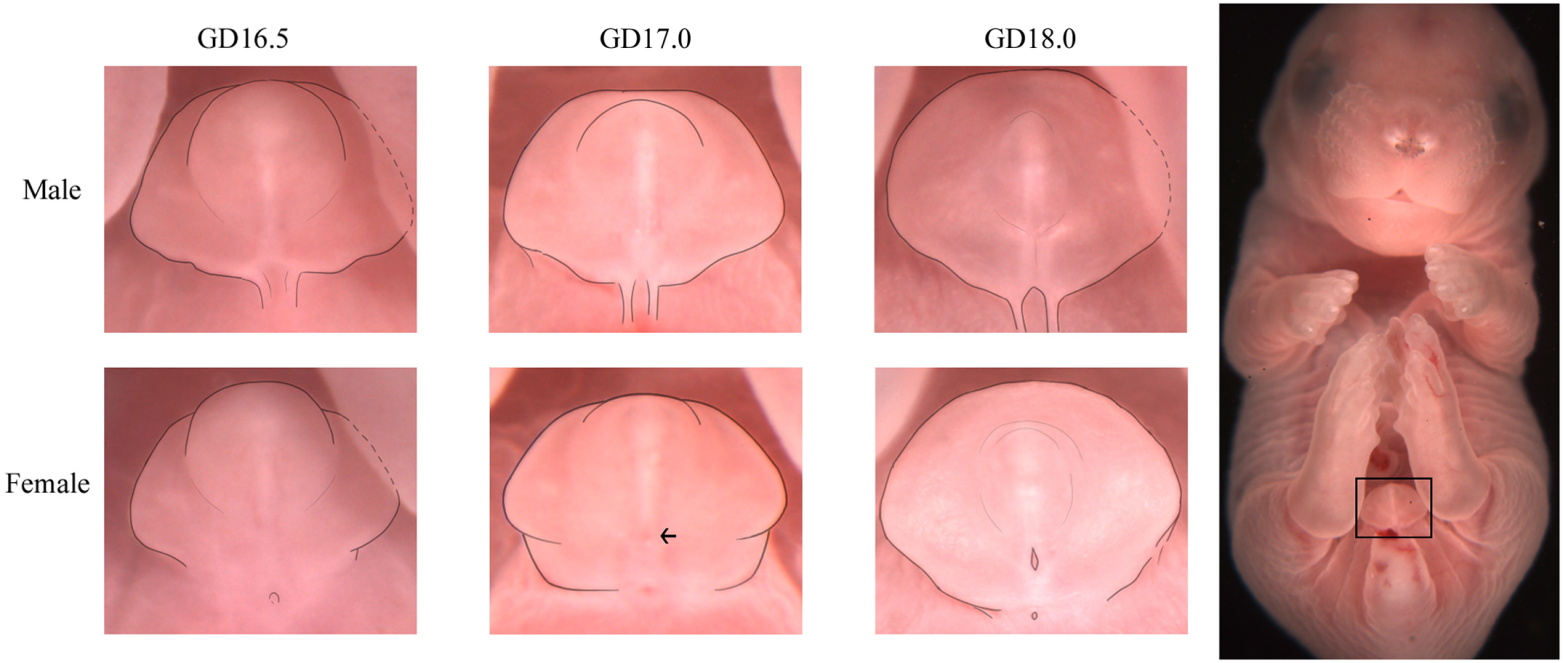
External genitalia development from GD 16.5 to GD 18.0 and body positioning. A timeline showing the growth of male *(top)* and female *(bottom)* external genitalia over a period of late gestation. Photographs of the genitalia are taken at approximately 1x, then cropped and lightly outlined for clarity. Hashed lines are used to indicate projected outline of the genitalia where an animals’ foot blocks it from view. Black arrow points to the small dark spot related to the urethral plate. *Right*, a full body photograph taken at approximately .90x demonstrates suggested positioning of the fetus for optimal examination (pictured animal is a GD 17.0 male).

External genitalia development begins around GD 11.5 when the genital swellings merge to become the genital tubercle. By GD 14.5 the external genitalia can be divided into three general areas on the ventral side (caudal to rostral); the scrotal/labial swellings, the preputial swellings which by GD 16.5 become the prepuce, and the distal glans (Fig 1) [14, 15]. Visible differences between the sexes begin to appear around GD 15.0, when the males display slightly larger scrotal swellings and a longer anogenital distance than do the females [14, 15]. However, without the use of precise measuring instruments, these features cannot accurately be used to determine sex at this day of development, and inconsistent lighting and positioning can further alter the appearance of these structures. Similarly, there are a number of common features between the sexes at GD 15.0, which makes distinguishing the sexes a challenge. These include the presence of a region related to the urethral plate (see Fig 2) which appears as a dark spot located approximately halfway between the glans and the base of the tubercle, and the proximal urethral meatus located at the base of the tubercle. This region is assumed to be related to the urethral plate, as its position and formation roughly align with that of the urethral plate and its eventual apoptosis forms the urethral opening in females, as summarized by Georgas et al (2015). Identifying fetal sex at GD 15.0 can also be challenging because any delay in the developmental rate would cause a fetus to be inspected prior to any significant differentiation. For these reasons, we recommend not using the methods described in this paper for fetuses prior to GD 16.5. By GD 16.5, enough differentiation has occurred that two critical features can distinguish the sexes with high accuracy, the urethral seam or urethral meatus, and the overall shape of the prepuce. On GD 17.0, the genitals have differentiated enough to be called the penis/clitoris, and an additional feature can be used to distinguish the sexes, this being the presence or absence of the region related to the urethral plate. However, this feature is much less reliable than the others and is not accurate in fetuses after GD 17.5. A timeline of growth for the male and female genitalia is shown in Fig 2, and a basic protocol-based flowchart to aid in sex determination is included.

## Methods

### Animals

The fetuses used in this study were generated from other ongoing projects within the lab in accordance with National Institutes of Health guidelines using methods and protocols approved by the Institutional Animal Care and Use Committee of the University of North Carolina (15–290).

C57BL6/J female mice (Jackson Labs, Bar Harbor, ME) were housed in groups of five or fewer, and breeder C57BL6/J males were single housed, in polycarbonate cages (39 x 20 x 16 cm) under controlled temperature and humidity with cob bedding and cotton nesting material, with free access to water and standard chow (Prolab Isopro RMH 3000, LabDiet, St. Louis, MO). Beginning about 4 hours into the light portion of a 12:12 light/dark cycle, 1–2 female mice (weighing at least 20g) were placed into a male’s cage for 1–2 hours. Females were then removed from the male’s cage and checked for the presence of a vaginal plug. GD 0 was defined as the beginning of the breeding period if a plug was found. Thus, 24 and 36 hours after the beginning of the breeding period would be GD 1 and 1.5, respectively.

### Fetal examination

Dams were sacrificed via CO_2_ asphyxiation followed by cervical dislocation. Fetuses were dissected out into ice-cold PBS, and then observed and photographed under a Nikon SMZ—U Stereoscopic Zoom dissecting microscope (Nikon Corporation, Melville, NY) with QCapture Suite software (QImaging, British Columbia, Canada). Dissection on GD 17.0 (n = 19 litters, 100 fetuses chosen) allowed for collection of age-appropriate, slightly developmentally delayed, and slightly developmentally advanced embryos from the same litters (approximately between GD 16.5 and GD 17.5). One litter taken at GD 16.5 (n = 8 fetuses) and another at GD 18.0 (n = 11 fetuses) were used to verify the applicability of the described sexing methods at the earlier and later developmental stages. After removal, fetuses were weighed and crown-rump length was measured. Each fetus was placed in a customized dish filled with ice-cold PBS to maintain the desired nearly prone position necessary to view the genitals (Fig 2). Tail samples were taken and stored in 70% ethanol at -20°c for subsequent PCR genotyping for the *Sry* gene. After photographs were taken as needed for the relevant study, fetuses were fixed in Bouin’s fixative for later analysis.

### *Sry* genotyping

In order to confirm sex in each fetus, tail samples were sent to an outside lab for PCR analysis. The *Sry* gene was amplified using forward primer 5’–TTG TCT AGA GAG CAT GGA GGG CCA TGT CAA—3’ and reverse primer 5’–CCA CTC CTC TGT GAC ACT TTA GCC CTC CGA– 3’. Amplification ran as follows: 3 minutes at 94°C, a 35x repeated cycle of 30 seconds at 94°C/ 30 seconds at 58°C/ 40 seconds at 72°C, followed by 2 minutes at 72°C and holding at 4°C. Samples were analyzed by agarose gel electrophoresis, and sex was determined based on the patterns of the bands present. The *Sry* locus ran at 273 bp.

### Photograph editing

**Fig 3:**
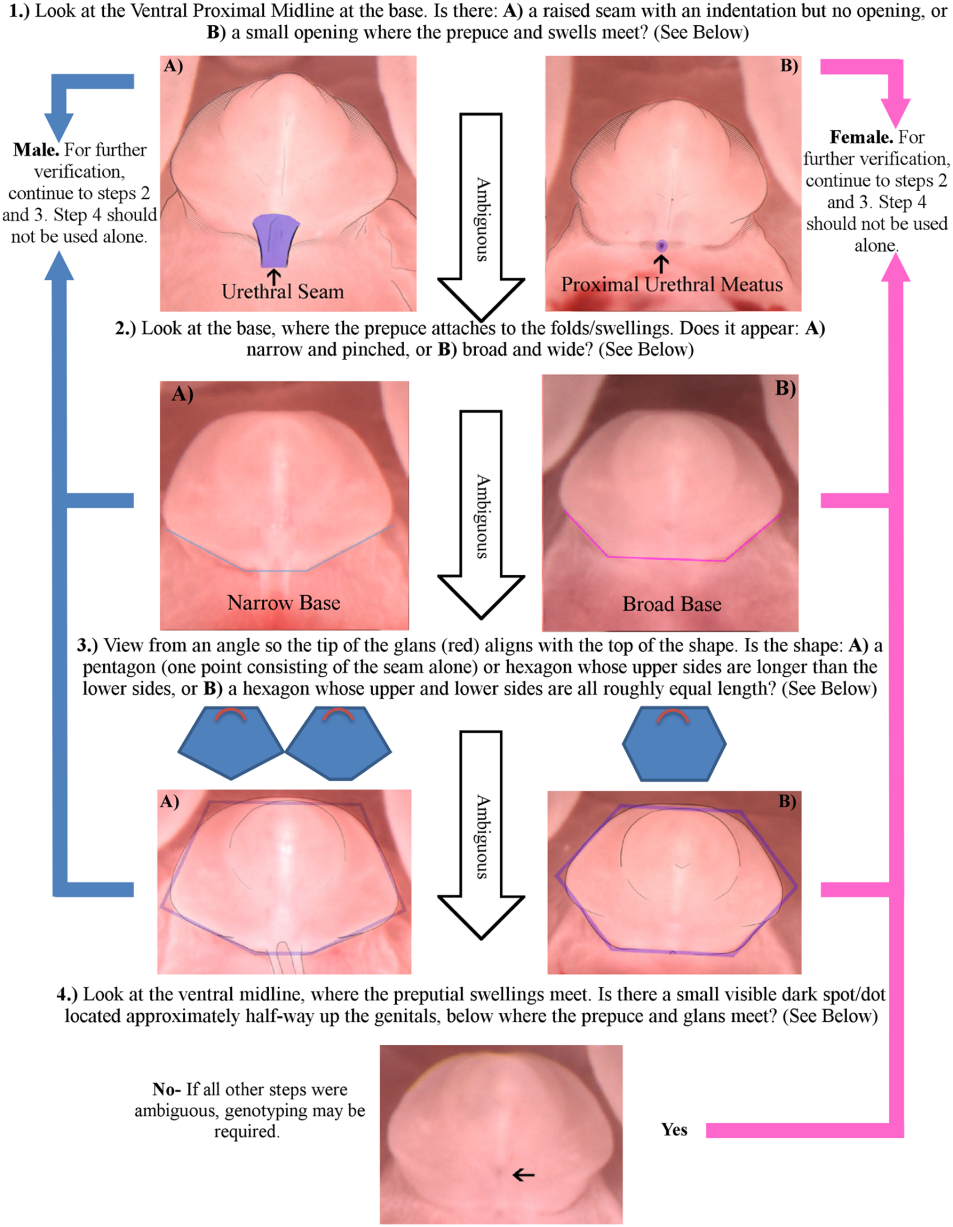
Flowchart style diagram of methods to sex GD 16.5 to GD 18.0 mice using the external genitalia. Steps are listed in decreasing order of effectiveness for mice at GD 17.0–17.5; Step 1 is the most effective, and step 4 is the least effective. Minor alterations to consider depending on the age of the fetus in question: 1) at GD 16.5 the dot related to the urethral plate (UP) may still be visible in some males, as the urethra is in the process of internalizing and so step 4 is not sex-specific; 2) after GD 17.5 the dot of step 4 is replaced with a slight opening, as seen in Fig 2 (GD 18.0 Female) which is sex-specific; 3) at GD 18.0 the preputial growth as occurred in males and females to the point that for steps 2 and 3 to be effective the glans must be positioned at least 3/4s of the way up the overall shape (as seen in Fig 2 GD 18.0 Male and Female). However, the envelopment of the glans makes it much more difficult to sex based on the dorsal half of the shape (step 3). It is suggested that at GD 18.0 one should thus focus on the angle or width of the base as opposed to the shape created with the upper sides.

To compare the effectiveness of single characteristics in determining sex, photos were edited using Adobe Photoshop Elements 5.0. The original photo was cropped and then edited into each of four categories; unedited full photo, Seam vs Meatus, genital shape and ventral midline. To isolate and emphasize each of these different anatomical characteristics all of the other identifying characteristics of the external genitalia were blacked out using the paintbrush tool at RGB (0,0,0). Seam vs Meatus photos were created by covering all of the tubercle except for a small portion of the base of the tubercle on the midline where it meets the labial/scrotal swellings, corresponding to the location of the proximal urethral meatus or urethral seam. Genital shape photos were created by blocking only the area of and around the ventral midline from approximately the labial/scrotal swellings to the tip of the glans. Ventral midline photos were created by blocking out all of the tubercle except for the ventral midline and adjacent space above the base of the tubercle, making sure to cover the seam or meatus. Examples of these can be seen in S1 Fig. The brightness, contrast levels, and color settings of the photographs used for the sexing test were not altered. As these photos were taken to test raters and the validity of the sexing method, a number of the photos are sub-optimal and reflect some of the challenges that can arise when examining a live fetus. These include instances where a foot/feet obscures part of the shape of the genitalia but leaves the midline and seam area free of obstruction (n = 40/100) and instances where blood obscures some of the genital base (n = 18/100) (for examples of these challenges see S2 Fig). The litters collected on GD 16.5 and GD 18.0 were only sexed using the full photograph method and via genotype. These litters were not included as part of the sexing test. Photographs (n = 100 per group) were examined one category at a time (e.g. all photos of isolated genital shape sexed, then all photos of isolated ventral midline sexed, etc.) with full photos rated last. Sexes were recorded and then hidden before moving on to the next group of photographs. Two initial raters who were blind to the sex of the fetuses, but had limited knowledge on the genital structures, were given an early version of the supplied flowchart (Fig 3) and completed the sexing test. Subsequently, two raters without any experience examining fetuses were given a draft of this paper and the supplied flowchart, and asked to complete the same sexing test to test the validity and ease of using the sexing protocol as written.

## Results and discussion

Examination of the external genitalia is an effective method for sexing fetal mice between GD 16.5 and GD 18.0, with high accuracy for both those with and without training. Determining the sex of a fetus relies on a number of sex-specific features and characteristics of the genitalia, and can be performed on fixed or unfixed fetuses as well as photographs. These features are discussed below in descending reliability as performed by raters with minimal experience, with exact numbers provided in the tables. While the presence of some of these features can accurately determine sex when used alone, it is suggested that a combination of features be used to determine sex as it increases the accuracy. Using the full, unedited photographs consistently produced higher accuracy and agreement rates than the most accurate single characteristic.

For clarity and ease of identification, we have included at the end of this paper a flowchart outlining the steps and methods for identifying the sex of an embryo at GD 17.0 (Fig 3) with visual aids. With a few modifications and caveats (given in the description of the figure) the flowchart can also be used to sex fetuses from GD 16.5 to GD 18.0.

### Seam vs meatus

**Fig 4:**
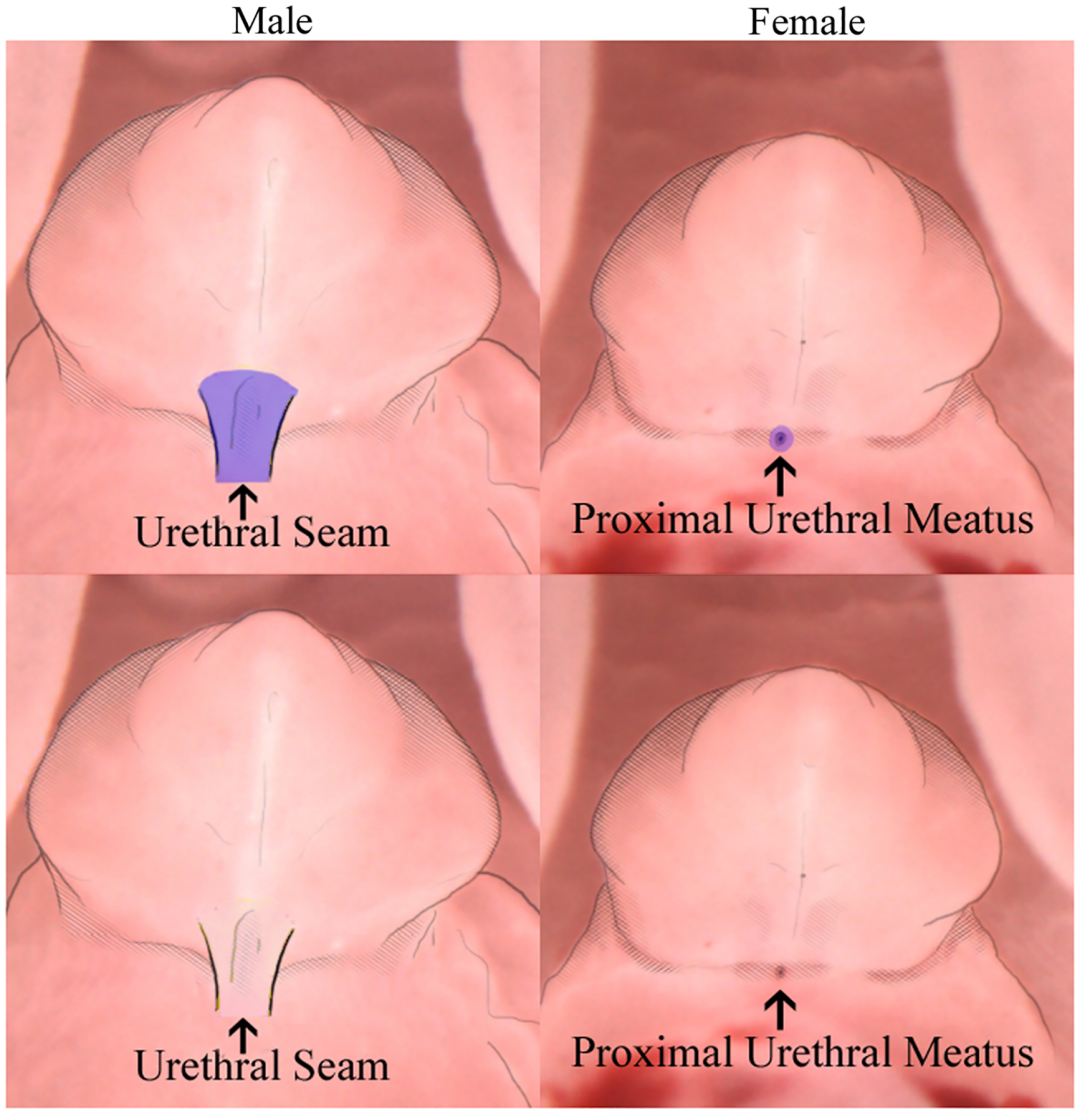
Sex specific physical characteristics of fetal mouse external genitalia. Photographs are of male *(left)* and female *(right)* external genitalia from a nearly ventral view at GD 17.0, and lightly outlined for visibility. *(Top)* Purple highlights the urethral seam and proximal urethral meatus. *(Bottom)* Highlights are removed for clarity and comparison.

The most distinctive sex-specific feature, and the most reliable single feature for sexing as performed by those with minimal training, can be found along the ventral midline at the base of the genital tubercle, corresponding to step 1 in Fig 3. Using photographs edited to show only a small portion of the base along the ventral midline, proved the most accurate single characteristic for determining sex at GD 17.0 for both examiners with minimal training and those without training. In this method, the fetus is examined for the presence of either a urethral seam or the proximal urethral meatus. Male fetuses (as confirmed by *Sry* genotyping) had a urethral seam at the base of their genital tubercles, while female fetuses (as confirmed by *Sry* genotyping) had an opening of the proximal urethral meatus instead (Fig 4). The urethral seam, also known as the urethral raphe or ventral seam, is a ridge of skin located at the base of the genital tubercle along the ventral midline, which traverses the crease where the scrotal folds meet with the body of the penis. This seam is the result of the canalization of the urethral tube within the penile body and the closing of the urethral meatus. The process occurs in a proximal-to-distal wave that is fully completed by GD 17.0 with the closing of the proximal urethral meatus in the males, but does not occur in females until postnatal (P) 8. By GD 17.0, the area related to the urethral plate located along the ventral midline approximately where the prepuce meets the glans is no longer visible in males, but is still visible as a dark spot in females [15]. In females, the urethra does not internalize and the area remains visible along the ventral edge of the genital tubercle until GD 17.5. The proximal urethral meatus, a small hole at the base of the genital tubercle which lacks any sort of raised skin that characterizes the male fetus, remains open at the base of the clitoris until P 8 when the area becomes the opening of the vagina [15].

Although the presence of a urethral seam or a proximal urethral meatus was the most accurate single physical characteristic for determining sex, using a combination of methods gave additional accuracy and agreement. Thus, the presence of a urethral seam or a meatus should not be the only factor used to determine sex. Furthermore, though infrequent in our study, cases of semi-feminized males and semi-masculinized females (S3 Fig) were observed, making sexing based on a single external characteristic ill-advised.

### Genital shape

Genital shape was also evaluated as an indicator of sex. Although not as distinctive as the urethral seam or proximal urethral meatus, genital shape alone was used to correctly predict the sex of an individual with nearly as much accuracy. Two ways of using the shape of the genitalia to determine the sex of a fetus are described and shown below. The first method primarily focuses on the proximal half of the genitalia, where the genital tubercle meets the labioscrotal swellings, while the second focuses on the top half of the structure. Our edited photographs used for testing showed the entire structure, which allowed raters to use either or both of the methods as needed.

**Fig 5:**
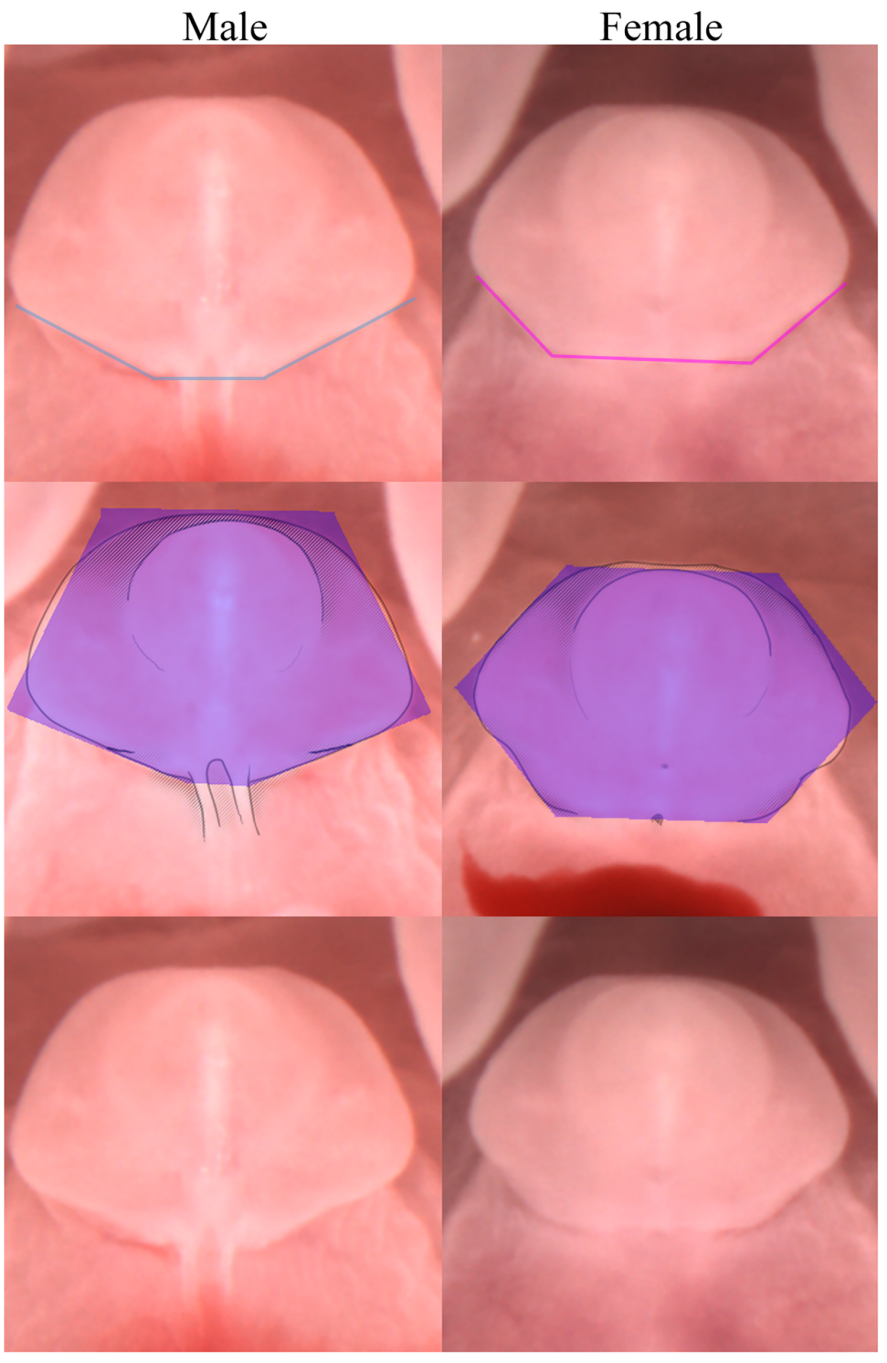
Differences in the width of the genital base and overall genital shape in males and females. Photographs of the male *(top*, *middle*, *and bottom left)* and female *(top*, *middle*, *and bottom right)* external genitalia from a nearly ventral view at GD 17.0. *Top*, the bases of the male *(left)* and female *(right)* external genitalia are lined to demonstrate their width. *Middle*, purple is used to illustrate the male’s resemblance to an irregular hexagon or pentagon, with upper sides longer than lower sides *(left)* and the female’s resemblance to a hexagon with upper and lower sides roughly equal in length *(right)*. *Bottom*, the lines and highlighting are removed so that the entire structure and base of the male *(left)* and female *(right)* can be viewed without obstruction.

The differences in genital shape arise due to a sex-based difference in the growth of the preputial swellings which begins at GD 15.0, as preputial swellings grow out further ventrally in males than in females [13, 14]. This growth difference leads to two related ways to identify fetal sex: the width and angle of the tubercle base and preputial swellings, or the overall shape of the organ (Fig 5). The first method focuses on the bottom half of the visible genital tubercle, where the tubercle meets with the labioscrotal swellings, while the second method puts more of a focus on the top half of the tubercle and the outline created by the dorsal side of the genital tubercle and preputial swellings. Our edited photographs used for testing showed both the top and bottom of the structure, which allowed raters to use either or both of the methods as needed.

To properly judge the sex based on the genital shape the fetus needs to be positioned nearly prone, although there is no single perfect angle. To create the smoothest outline the ventral side of the genital should be fully visible, and the tip of the glans should roughly align with the outline of the dorsal side, located fully within the area of the prepuce (examples of this can be seen in Figs 3 and 5). In this position the top and bottom of the genital shape is created by the dorsal side of the tubercle, where the tubercle meets with the swellings. Looking at the bottom half of the genital shape, the male’s base consists of a narrow area where the urethral seam joins with the main body of the genitals, as the increased preputial outgrowth creates a visually distinct overhang and narrows the portion that appears in direct contact with the swellings (Fig 5 *top left*). In contrast, the female’s base runs the entire length of where the tubercle meets with the labial swellings, and is significantly longer than the males base (Fig 5 *top right*). The lack of preputial growth in females also means that the internal angle created by the sides of the structure/swellings meeting with the base will be smaller (~140°) as compared to the narrower male base (~150°).

Looking at the top half and overall shape of the tubercle, the genital structure of the male and female resemble geometric shapes, with the top of the shape created by the dorsal side of the tubercle, and the bottom created by the area where the tubercle meets with the labioscrotal swellings. The outline created by the penis will resemble an irregular hexagon with one vertical line of symmetry, with the upper left and right sides noticeably longer than the lower left and right sides, and the genital base being the shortest of all sides (Fig 5 *middle left*). By GD 18.0 continued preputial growth means the male genital outline looks more like a pentagon, with the downward facing point created by the urethral seam, with upper sides noticeably longer than any of the other sides. The outline created by the clitoris will resemble a hexagon with two lines of symmetry, whose longest sides are the horizontal top and bottom, and whose other sides are roughly equal in length (Fig 5 *middle right*). One advantage to using the shape method to sex is that an individual can be sexed in the event that the area around the urethral seam or proximal meatus becomes obscured.

### Ventral midline

**Fig 6:**
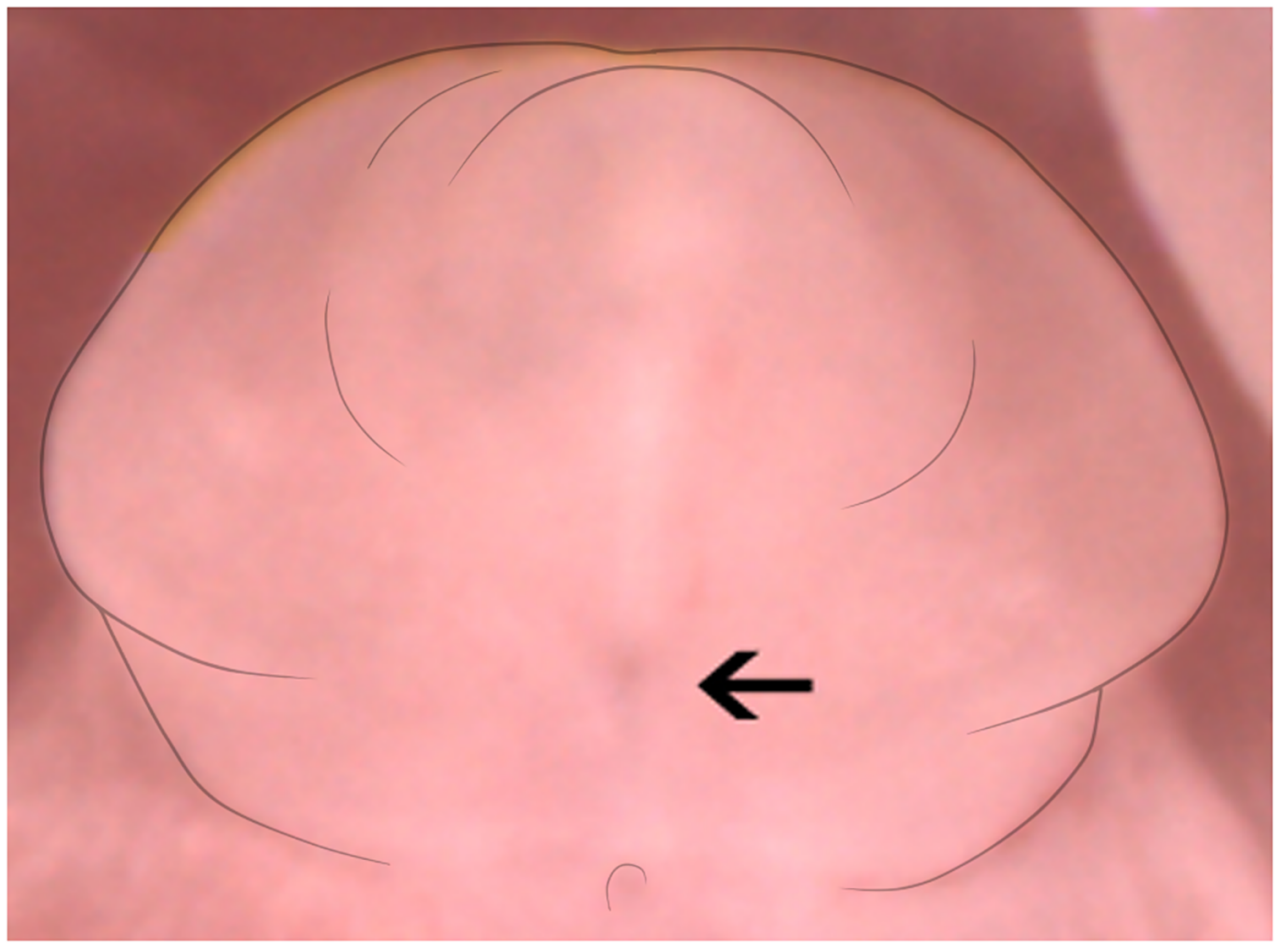
Example of spot related to the urethral plate in GD 17.0 female mouse. The black arrow indicates the location of a dark patch visible in the female genitalia, related to the urethral plate.

Attempting to sex fetuses at GD 17.0 solely based upon the presence of the small dot along the ventral midline related to the urethral plate proved the least accurate single characteristic to determine sex. While it is a sexually dimorphic feature, the appearance of a dark spot related to the urethral plate (Fig 6) is not useful by itself as a tool to determine sex; lighting and positioning can make it blend in with the rest of the tubercle, and it is only visible in females until GD 18.0, when the region becomes the dorsal urethral opening. The urethral plate related dark spot can be used in combination with other methods to determine the sex of a fetus, such as the presence or absence of a urethral seam and the shape of the base, but it should not be the primary factor for determining the sex of a fetus. Another disadvantage of attempting to sex based on the urethral plate area is that, depending on the preservation method used, it may not be visible in post-hoc analyses.

### Methodological accuracy

For raters with minimal experience examining fetal mouse genitalia the use of multiple characteristics to determine the sex of an individual proved the most accurate, with an average accuracy rate of 99.5% and 98% interrater agreement (Table 1). The most accurate single characteristic for identifying sex was the presence of a urethral seam or a urethral meatus, with an average 96% accuracy and 92% interrater agreement. The next most accurate single characteristic for identifying sex was still highly accurate, but provided only an average 92.5% accuracy and 93% interrater agreement. The least accurate feature for identifying sex was looking for the presence of the small dark spot related to the urethral plate, which yielded only an average 62% accuracy rate and 53% interrater agreement.

**Table 1:** Number of animals correctly sexed using external genitalia by raters with minimal experience.

	Rater 1 # Correct (Out of 100)	Rater 2 # Correct (Out of 100)	Average # Correct	# Agree Between Raters
Full Photo	100	99	99.5	98
Seam vs Meatus	98	94	96	92
Genital Shape	92	93	92.5	93
Ventral Midline	72	52	62	53

Number of pups correctly sexed using external methods (as compared to genotypic sexing) by examiners with minimal experience examining fetal mouse genitalia, as well as interrater agreement.

Raters with no experience performed similarly, with the full photograph and combination of features proving the most accurate method of sexing a fetus, with a 95% average accuracy, and 95% interrater agreement (Table 2). However, when performed by raters without any experience sexing fetal mice, the most accurate single characteristic to determine sex was found to be overall genital shape (average 93% accuracy, 94% interrater agreement) rather than the presence of a urethral seam or urethral meatus (average 89% accuracy, 90% interrater agreement). Sexing based on the presence or absence of the small dot related to the urethral plate proved the least accurate, with an average 61.5% accuracy and 77% interrater agreement.

**Table 2:** Number of animals correctly sexed using external genitalia by raters with no experience.

	Rater 3 # Correct (Out of 100)	Rater 4 # Correct (Out of 100)	Average # Correct	# Agree Between Raters
Full Photo	94	96	95	95
Seam vs Meatus	85	93	89	90
Genital Shape	92	94	93	94
Ventral Midline	63	60	61.5	77

Number of pups correctly sexed using external methods (as compared to genotypic sexing) by examiners with no experience examining fetal mice, as well as interrater agreement.

## Conclusion

Here, we demonstrate that the external characteristics of the genitalia provide a reliable and fast method for determining sex in GD 16.5—GD 18.0 mouse fetuses. Though this method was tested largely in C57BL/6J mice, because it is based on basic morphological features, it could also be used in albino or lightly pigmented mice. We tested this method in several litters of B6/129S4 mice, which present with either black or agouti coloring, with 100% accuracy. Using this method, sex can be determined from freshly dissected or fixed fetuses, or from still photos, making it possible to examine previously collected samples for sex-specific effects. This technique is based on physical characteristics, and requires only a dissecting microscope, forceps, and a receptacle to hold the fetuses. Incorporating these methods into our current dissection protocol has added a negligible amount of time to our normal photographing procedures, and has a near 100% accuracy. After removing the tail and positioning the fetus (a process which typically takes about a minute per fetus), an experienced rater can determine sex within seconds. Once a rater has become sufficiently familiar with the various features that distinguish the sexes, sexing can be accurately performed even in cases where the genitals are obscured or the fetus is improperly positioned. Included as supporting information is a set of 50 numbered photographs of unobscured genitalia and a key as to their sex which can be used to practice sexing (S1 File). With experience, raters will become more familiar with the external anatomy, improve their accuracy and no longer require the diagram.

## Supporting information

S1 Fig Examples of photograph editing for genital characteristic isolation. Figure shows example of the methods used to isolate and emphasize the anatomical characteristics for the purpose of testing sex based on physical appearance. All photographs are of one male individual. A shows the unedited full photo, free of any markings. These were used in the “Full Photo” category of testing. B shows an example of the editing used to isolate the genital shape, by blocking both the ventral midline and the genital base. These were used in the “Genital Shape” category of testing. C shows an example of the editing used to isolate the ventral midline, by blocking all genital characteristics except for the ventral midline. These were used in the “Ventral Midline” category of testing. D shows an example of the methods used to isolate the ventral midline of the genital base, by blocking all characteristics of the genitalia except for the portion of the genital base corresponding to the location of the urethral seam or proximal urethral meatus. These were used in the “Base” category of testing.
(TIF)

S2 Fig Examples of sub-optimal test photos reflecting challenging factors. Both photographs are of males that were not included in the sexing test. *Left* is an example of a foot blocking a portion of the genitalia, which was present in 40 out of the total 100 photographs for each group of the sexing test. *Right* is an example where both feet are blocking part of the genitalia as well as the presence of some blood obscuring the genital base and seam. The obscuring occurred in 17 of the total 100 photographs per group.
(TIF)

S3 Fig Example of semi-masculinized female, and semi-feminized male. Dashed black lines are used to project the shape of the genitalia when part of the foot obstructs it. The lower portion of the genitalia is left unlined to demonstrate the indeterminate nature of the structures. *Left* is an example of a semi-masculinized female. Due to the angle of the photograph and light blood pooling around the base it is difficult to classify it as a broad or narrow base. It is also difficult to see the presence of a definitive urethral meatus. The structure also appears to have a small urethral seam, though it does not appear as raised as the typical male urethral seam. Subsequent genotyping revealed this to be a female, despite the semi-masculine appearance. *Right* is a semi-feminized male. This fetus has outgrowth of the preputial swellings that is not as well-defined as other males (blue dashed lines). The lack of preputial outgrowth makes the genital base not as narrow as other males. The urethral seam is also not as raised as a typical male urethral seam, and is indented. Subsequent genotyping revealed this to be male, despite the indeterminate features. As we have continued visually sexing pups for our ongoing studies we have found such indeterminate genitalia to be a rare occurrence. There have been approximately 7 cases out of approximately 520 fetuses where we assigned a sex but felt the need to genotype due to abnormal genital appearance. Of those 7 (which included males and females) only 1 had been incorrectly assigned a sex.
(TIF)

S1 File Test set of 50 practice photos and answer key. Set of 50 numbered photographs of genitalia on GD 17.0, and key to their sex. Photos are near optimal, with little to no instances of feet or blood obscuring any features, making them ideal for training.
(PDF)
