# Supplementary figures and images for: A novel method for determining sex in late term gestational mice based on the external genitalia
Source: PLoS One. 2018 Apr 4;13(4):e0194767. doi: 10.1371/journal.pone.0194767 (PMC5884523; doi:10.1371/journal.pone.0194767)

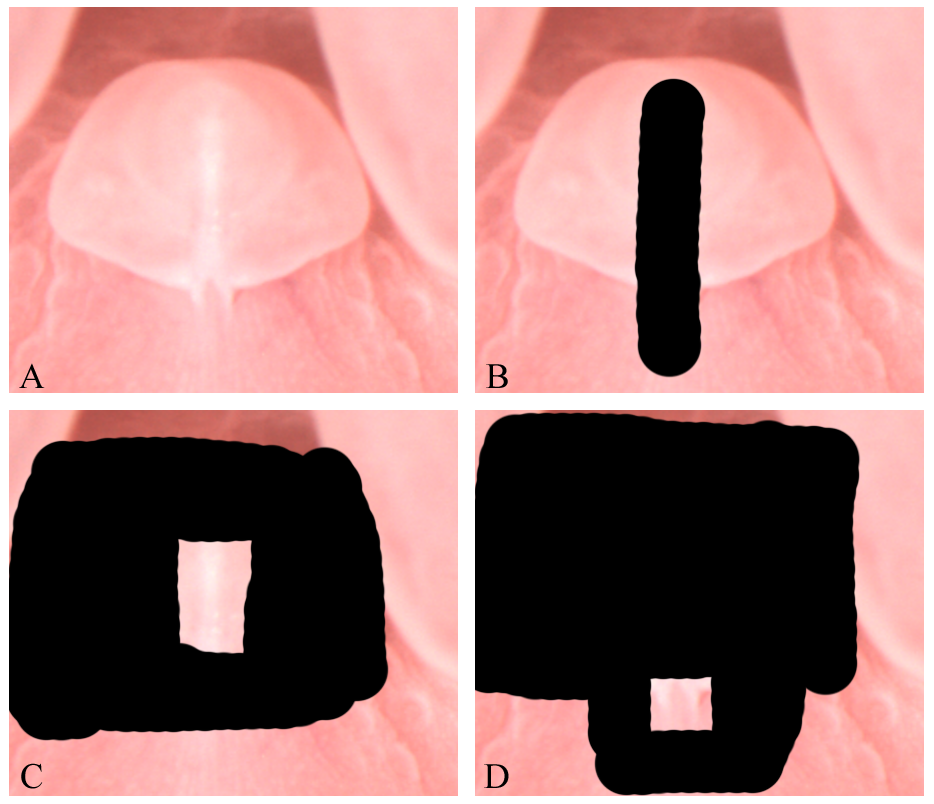

Supplement: S1 Fig — Figure shows example of the methods used to isolate and emphasize the anatomical characteristics for the purpose of testing sex based on physical appearance. All photographs are of one male individual. A shows the unedited full photo, free of any markings. These were used in the “Full Photo” category of testing. B shows an example of the editing used to isolate the genital shape, by blocking both the ventral midline and the genital base. These were used in the “Genital Shape” category of testing. C shows an example of the editing used to isolate the ventral midline, by blocking all genital characteristics except for the ventral midline. These were used in the “Ventral Midline” category of testing. D shows an example of the methods used to isolate the ventral midline of the genital base, by blocking all characteristics of the genitalia except for the portion of the genital base corresponding to the location of the urethral seam or proximal urethral meatus. These were used in the “Base” category of testing. (TIF) [file pone.0194767.s001.tif]

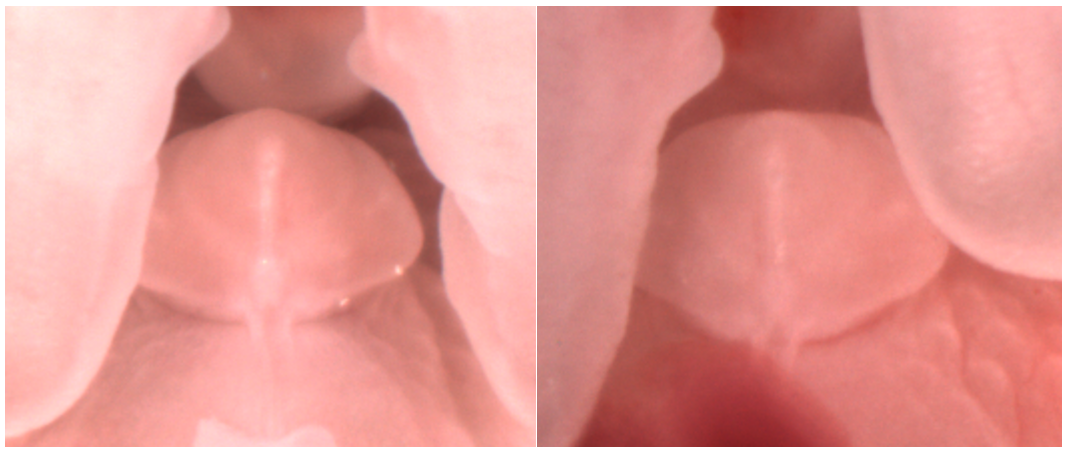

Supplement: S2 Fig — Both photographs are of males that were not included in the sexing test. Left is an example of a foot blocking a portion of the genitalia, which was present in 40 out of the total 100 photographs for each group of the sexing test. Right is an example where both feet are blocking part of the genitalia as well as the presence of some blood obscuring the genital base and seam. The obscuring occurred in 17 of the total 100 photographs per group. (TIF) [file pone.0194767.s002.tif]

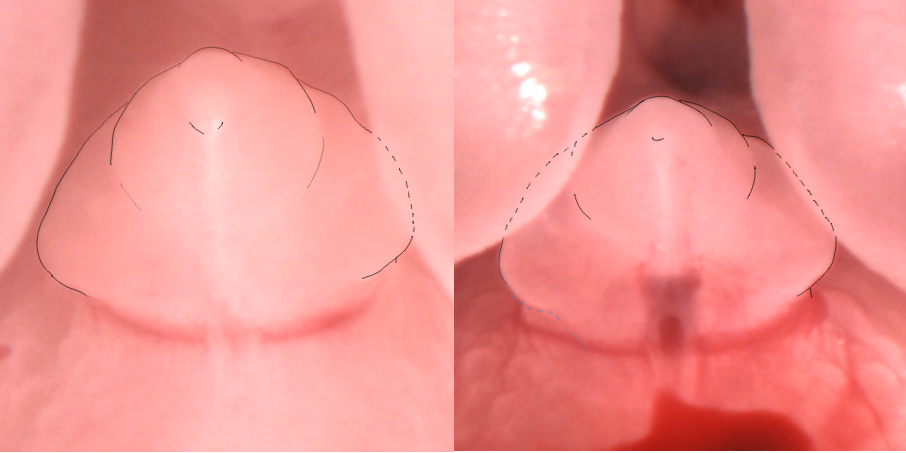

Supplement: S3 Fig — Dashed black lines are used to project the shape of the genitalia when part of the foot obstructs it. The lower portion of the genitalia is left unlined to demonstrate the indeterminate nature of the structures. Left is an example of a semi-masculinized female. Due to the angle of the photograph and light blood pooling around the base it is difficult to classify it as a broad or narrow base. It is also difficult to see the presence of a definitive urethral meatus. The structure also appears to have a small urethral seam, though it does not appear as raised as the typical male urethral seam. Subsequent genotyping revealed this to be a female, despite the semi-masculine appearance. Right is a semi-feminized male. This fetus has outgrowth of the preputial swellings that is not as well-defined as other males (blue dashed lines). The lack of preputial outgrowth makes the genital base not as narrow as other males. The urethral seam is also not as raised as a typical male urethral seam, and is indented. Subsequent genotyping revealed this to be male, despite the indeterminate features. As we have continued visually sexing pups for our ongoing studies we have found such indeterminate genitalia to be a rare occurrence. There have been approximately 7 cases out of approximately 520 fetuses where we assigned a sex but felt the need to genotype due to abnormal genital appearance. Of those 7 (which included males and females) only 1 had been incorrectly assigned a sex. (TIF) [file pone.0194767.s003.tif]

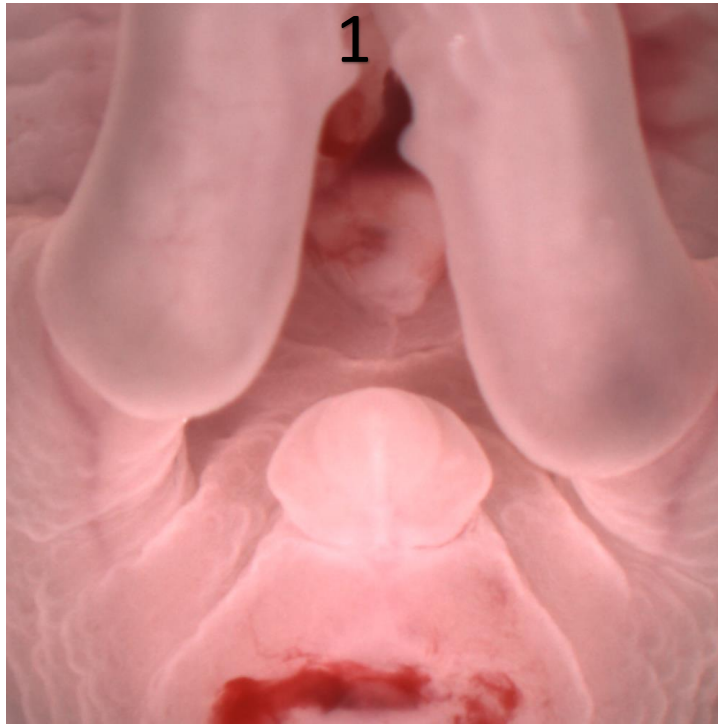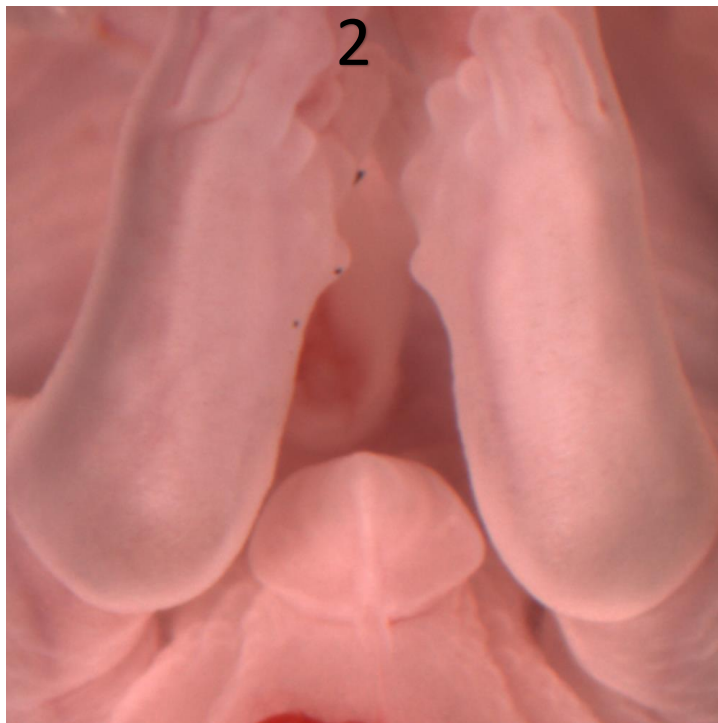

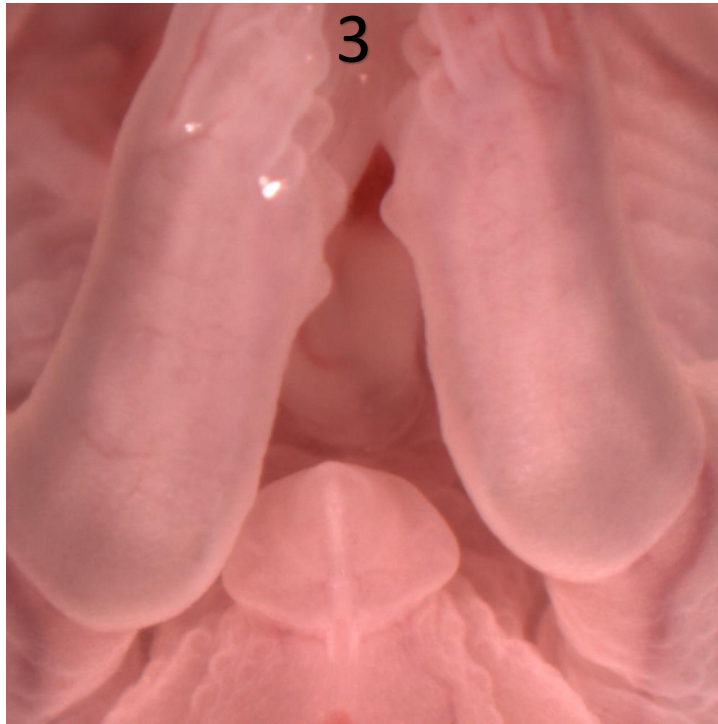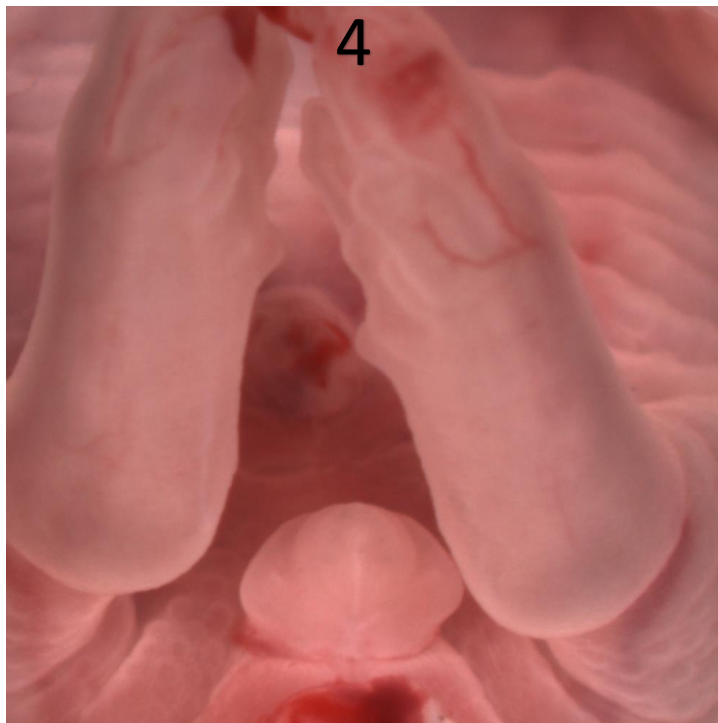

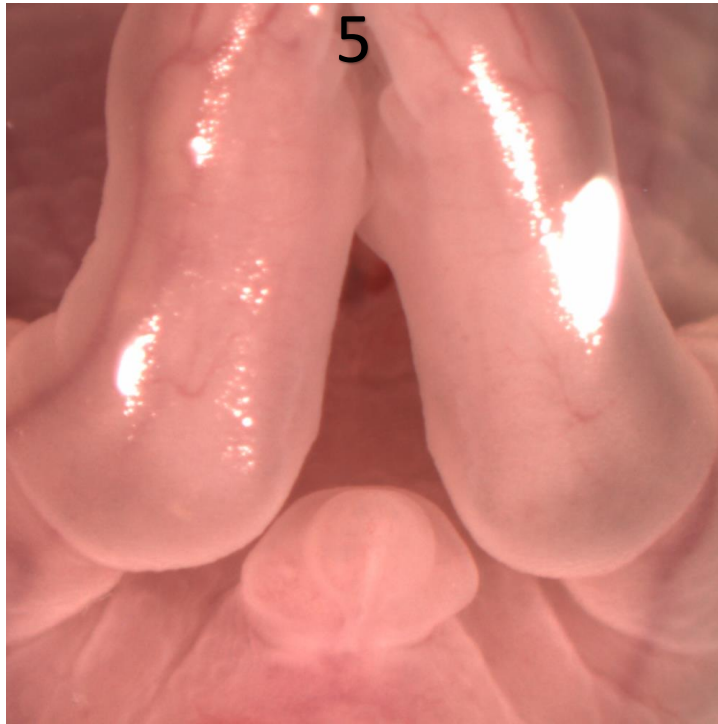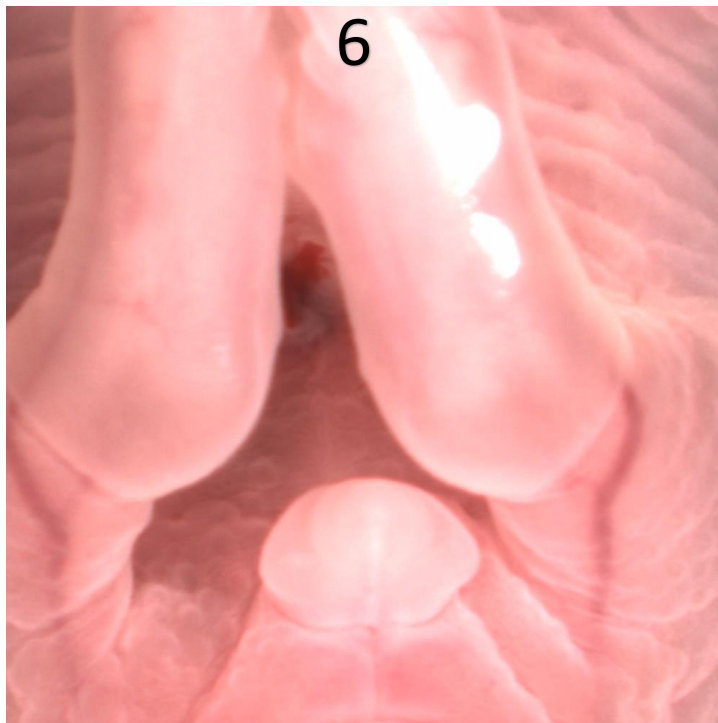

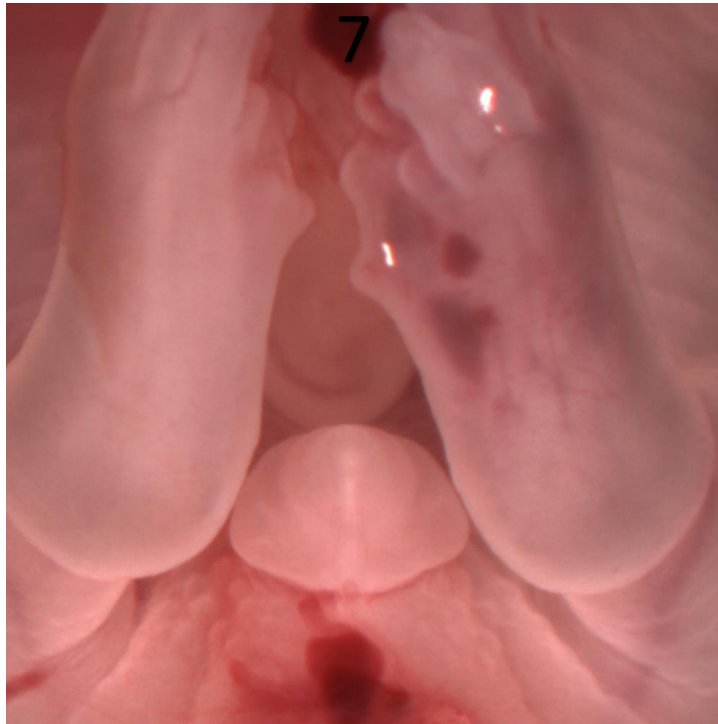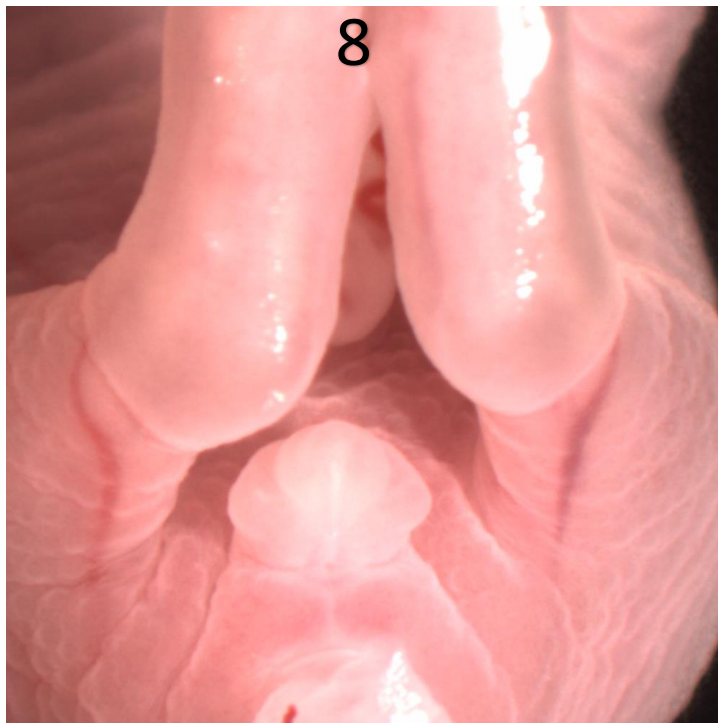

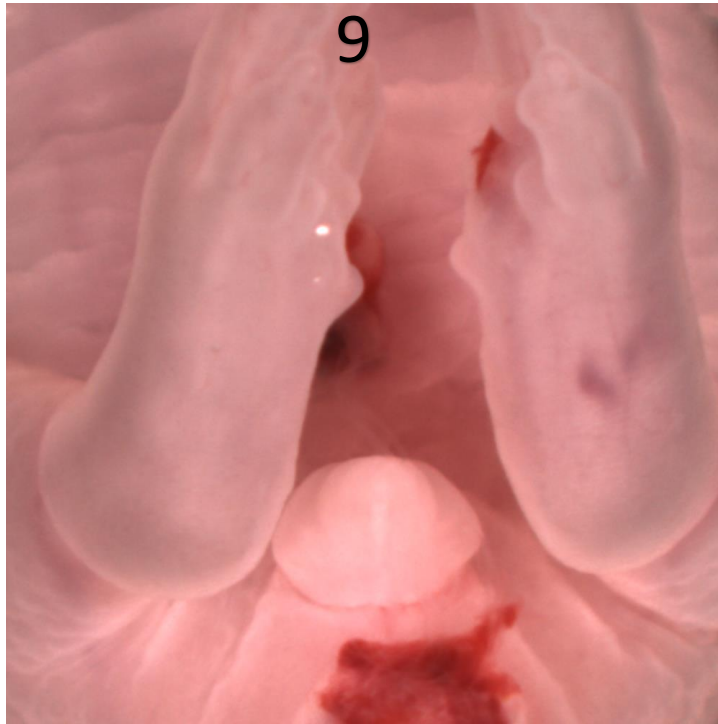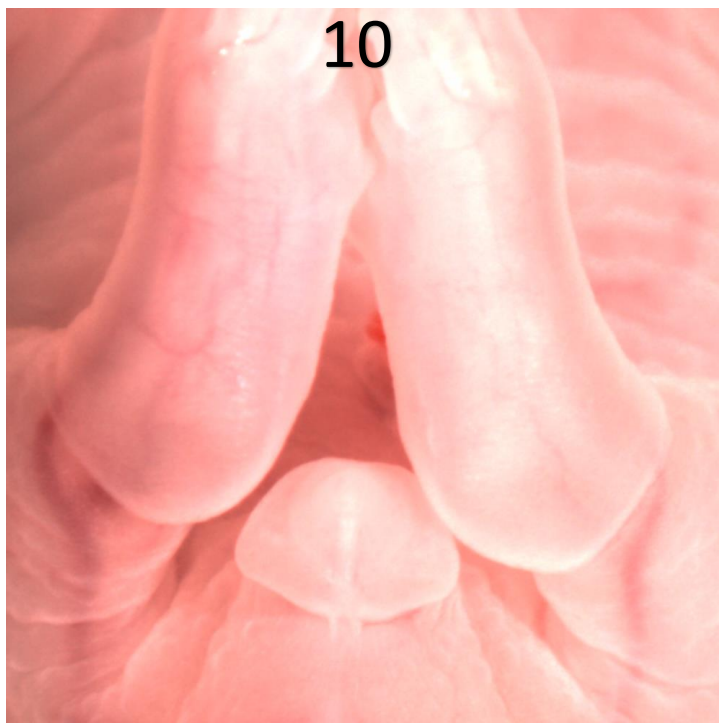

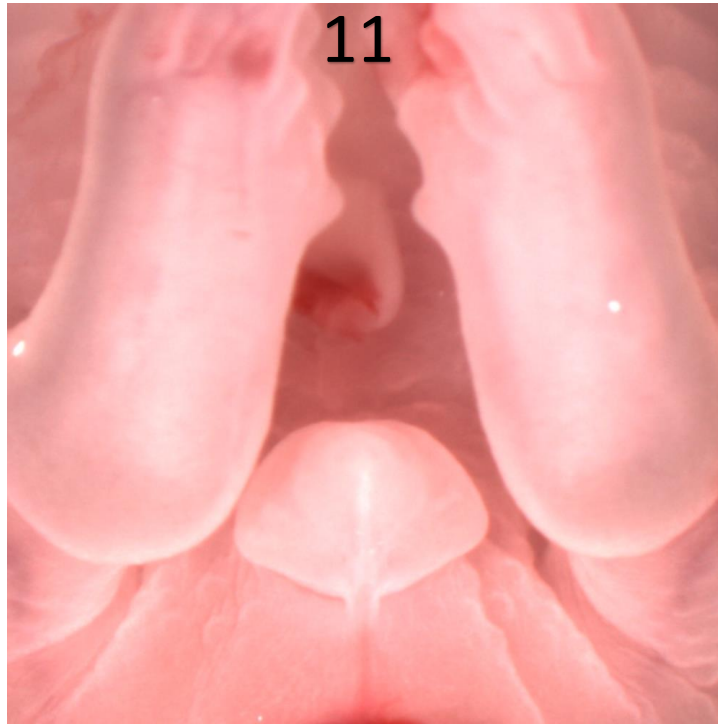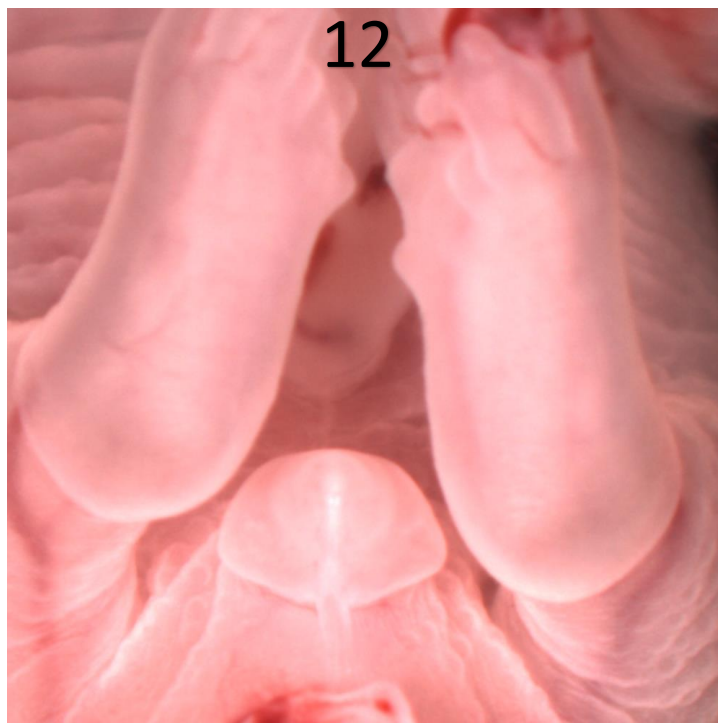

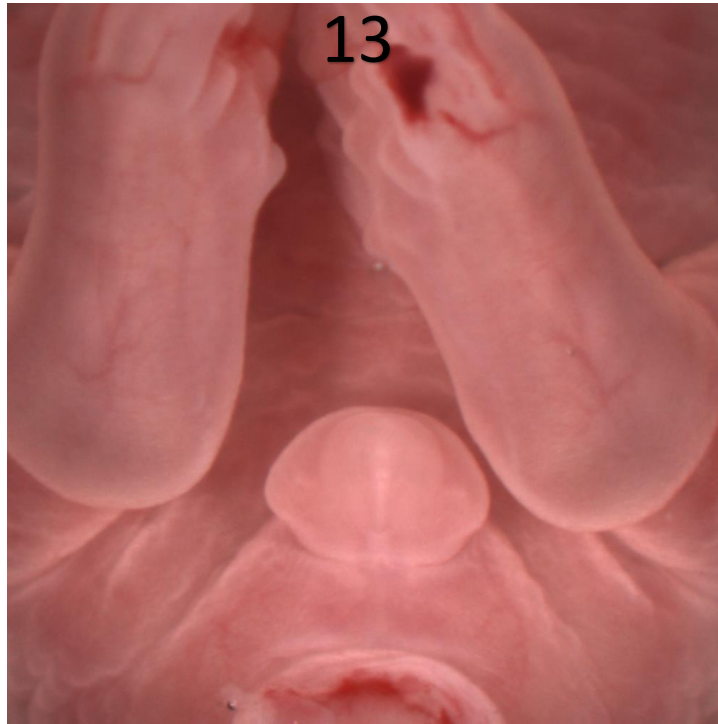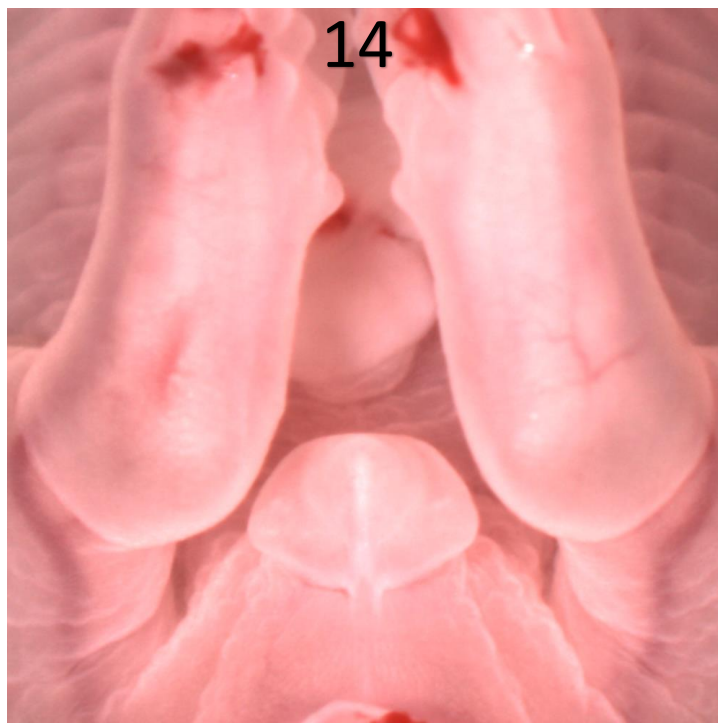

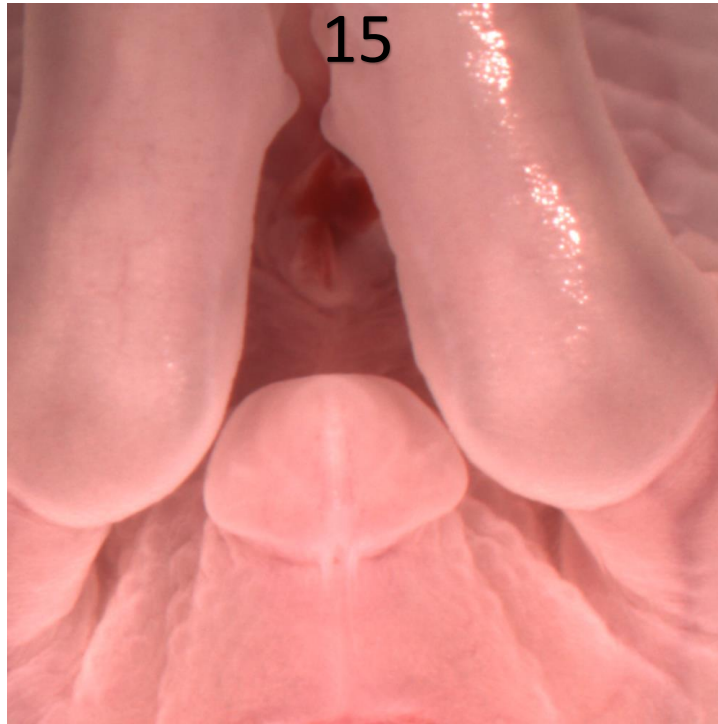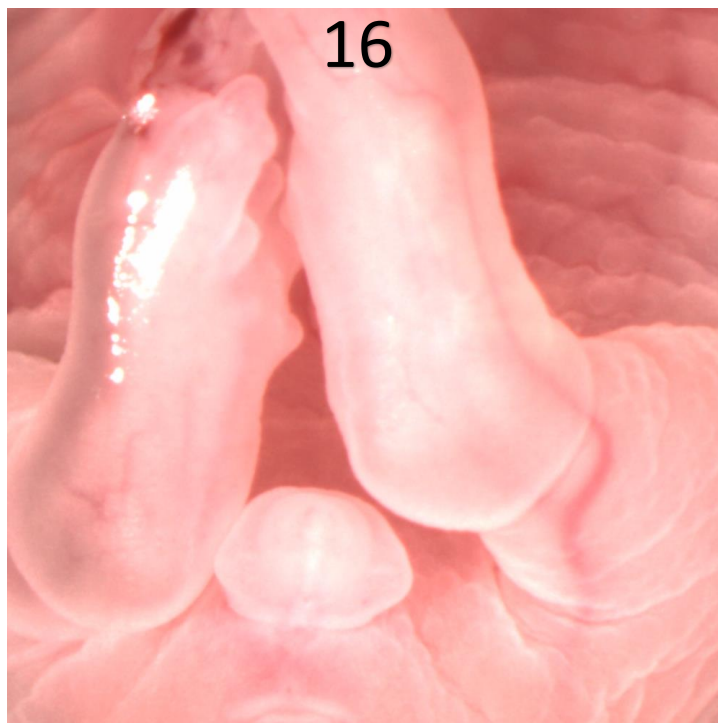

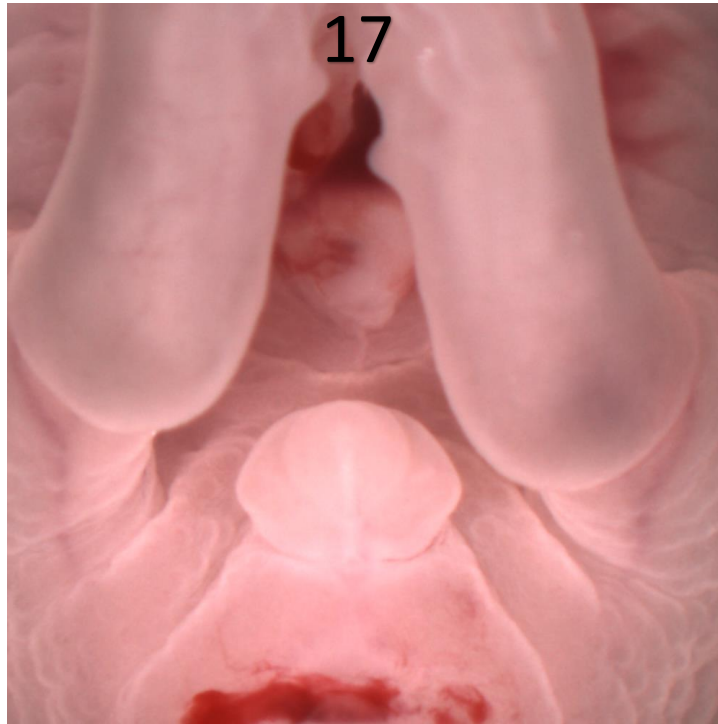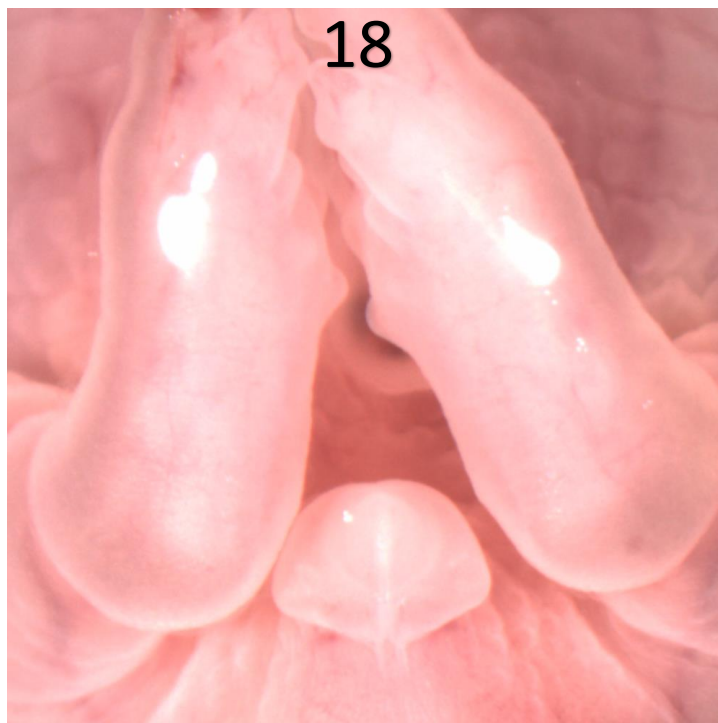

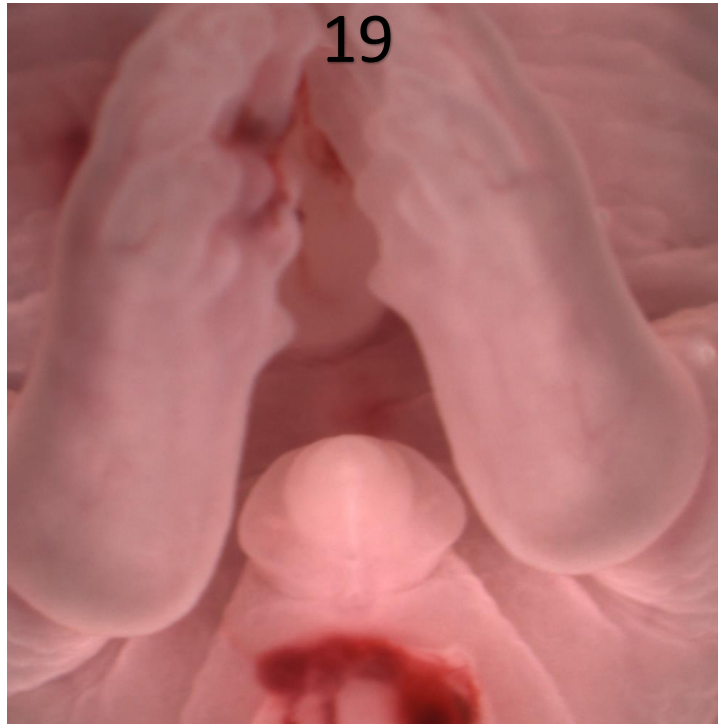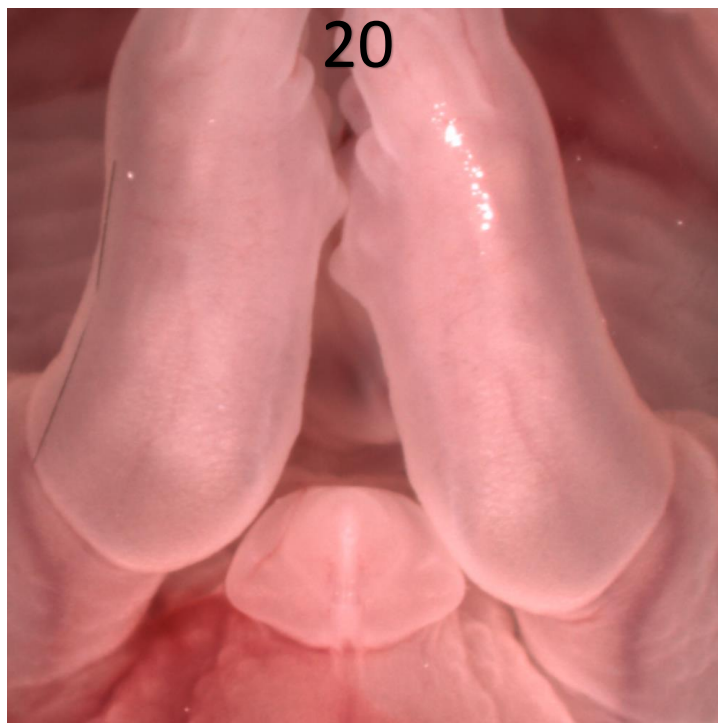

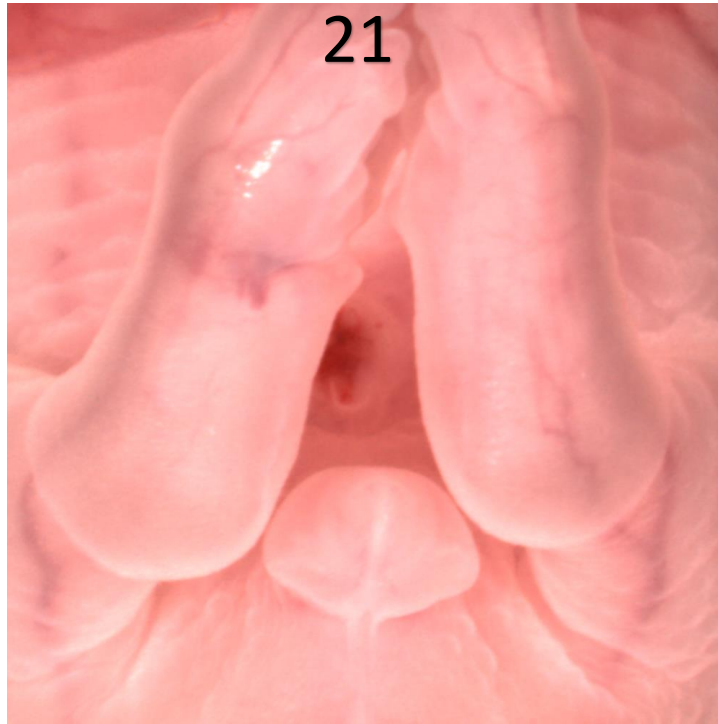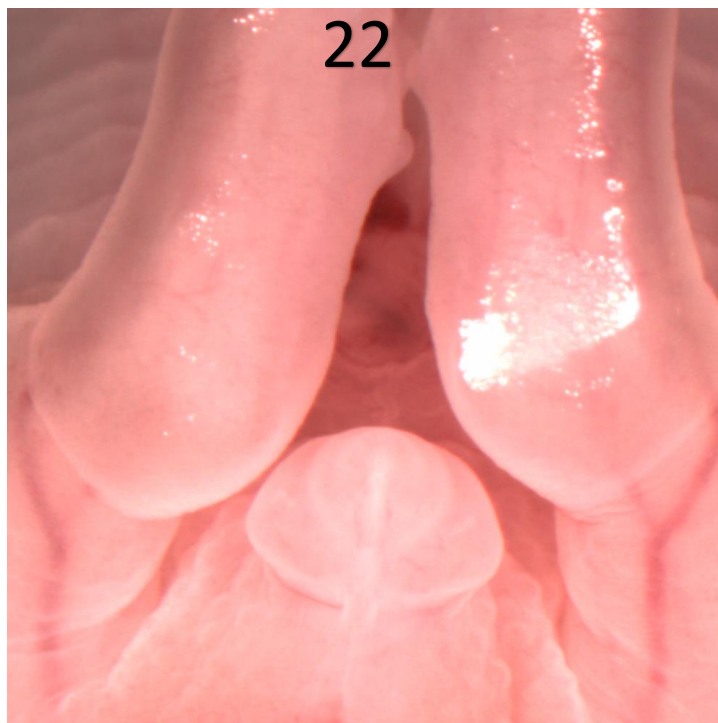

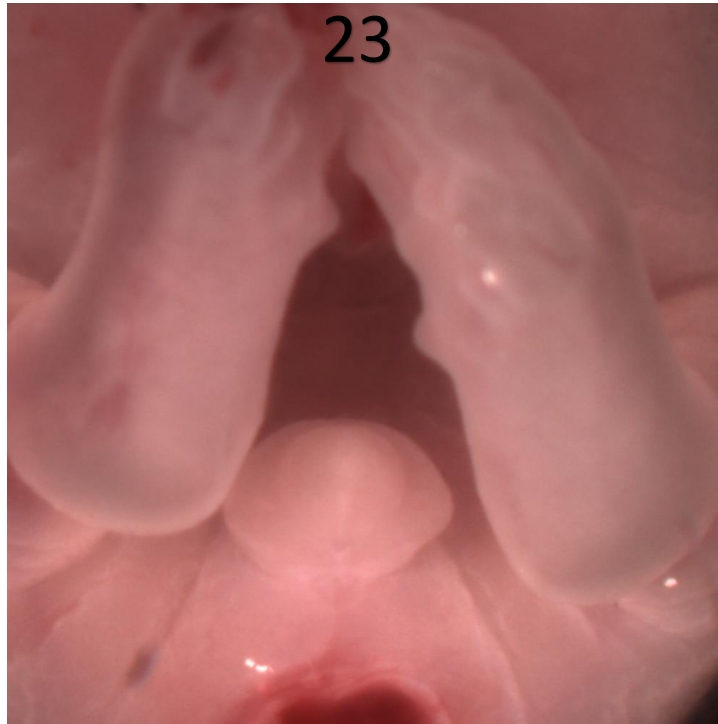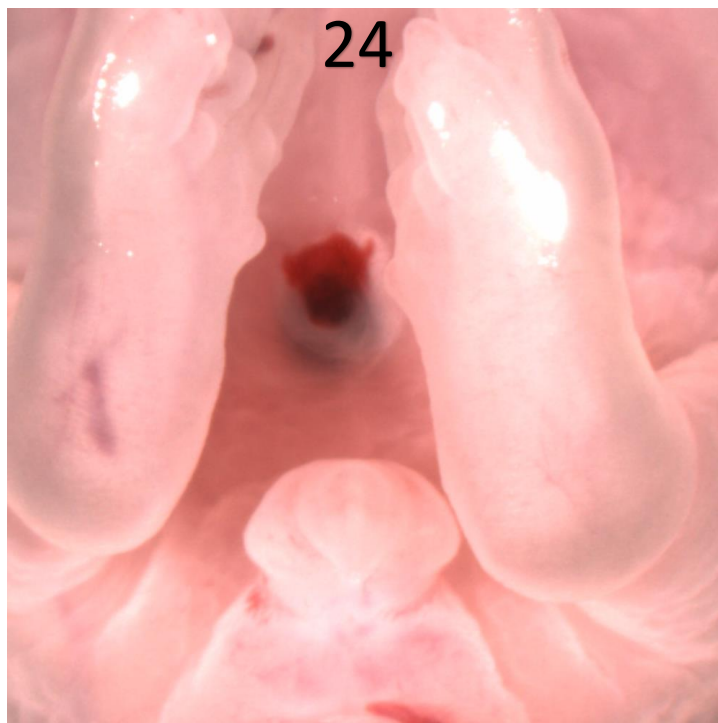

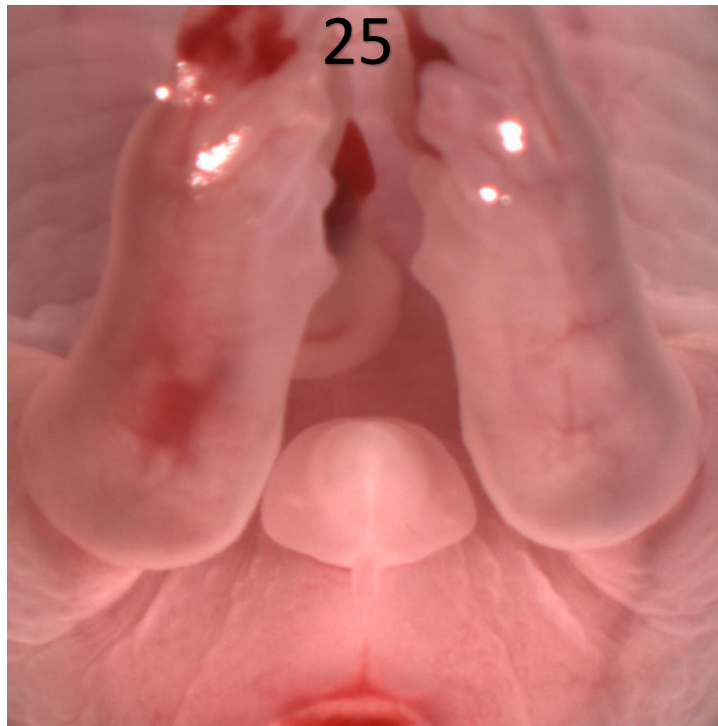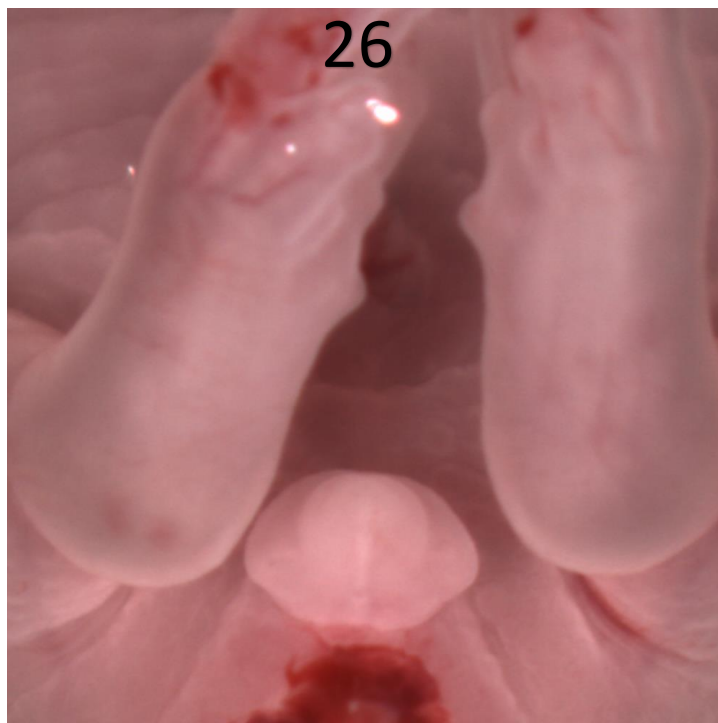

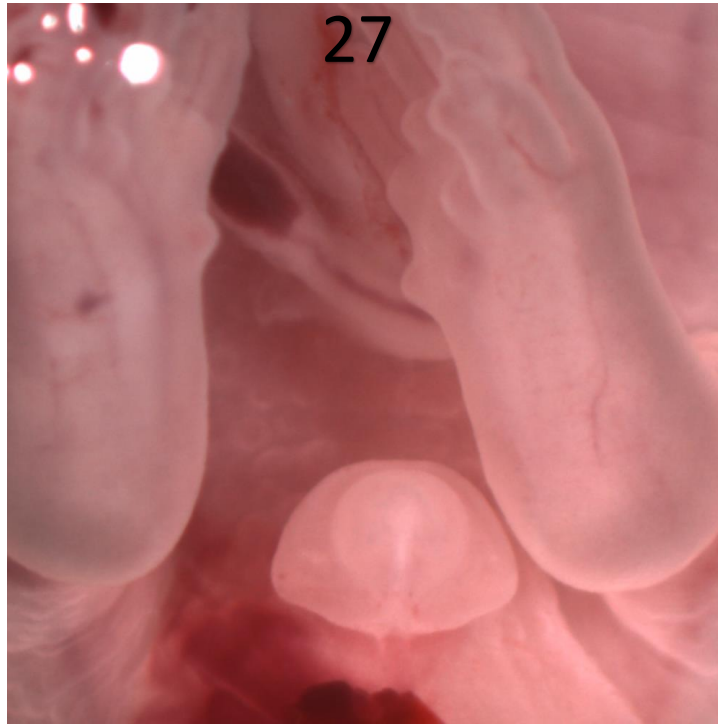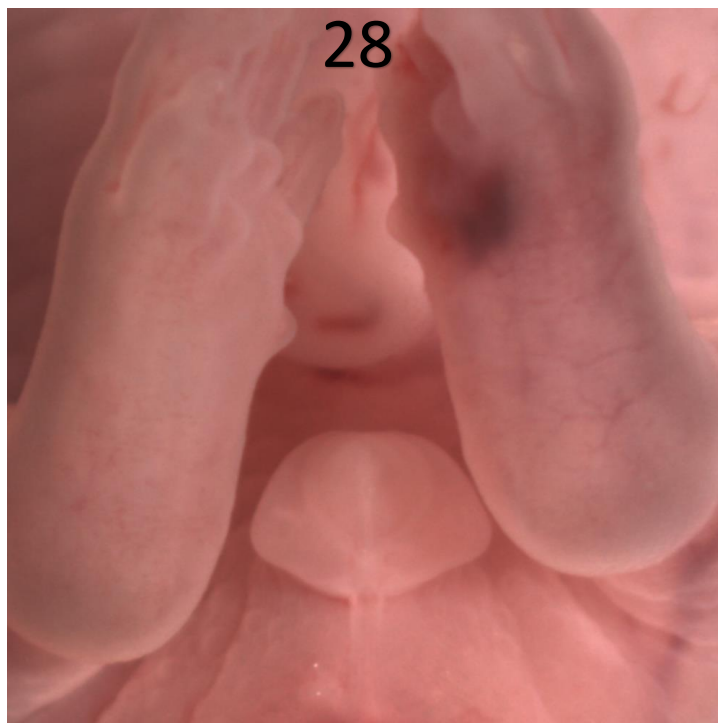

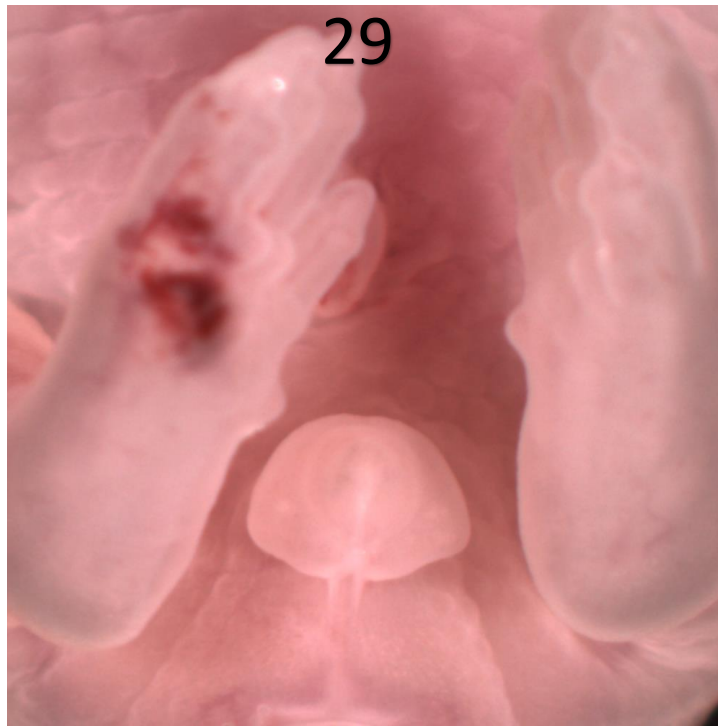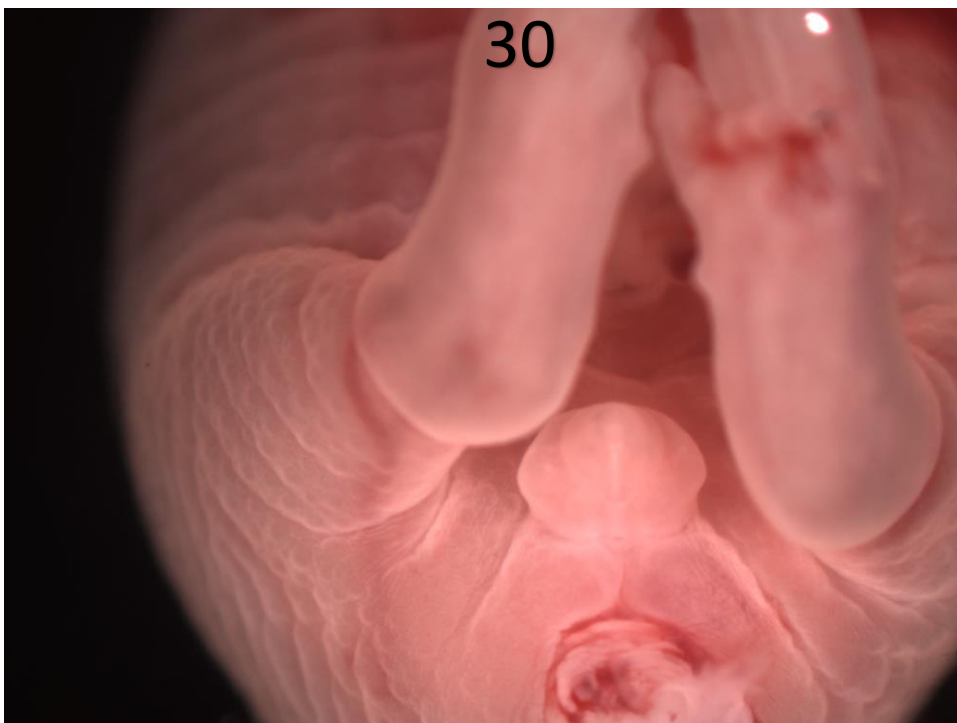

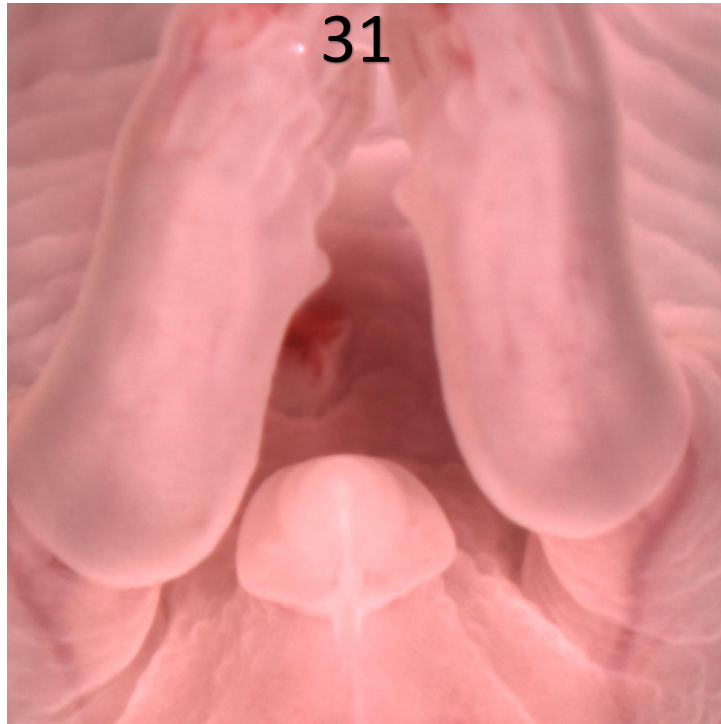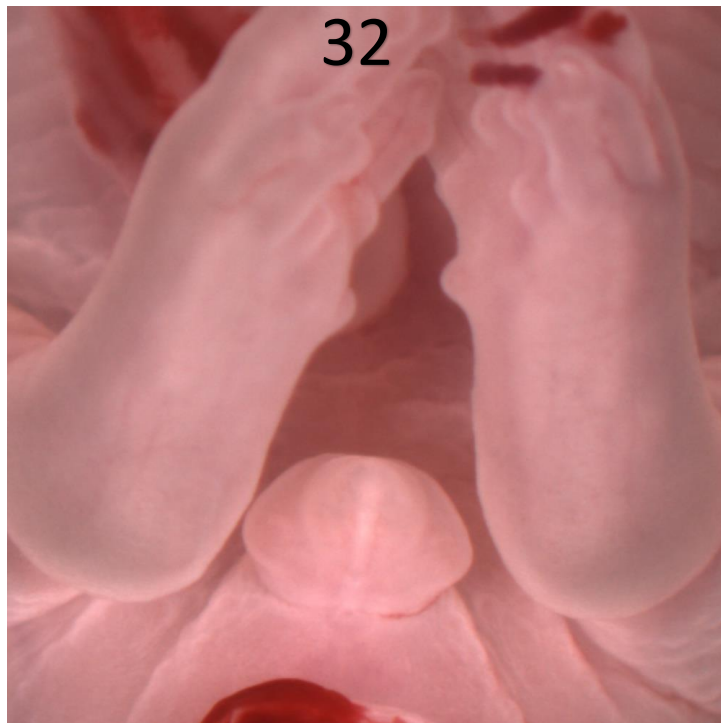

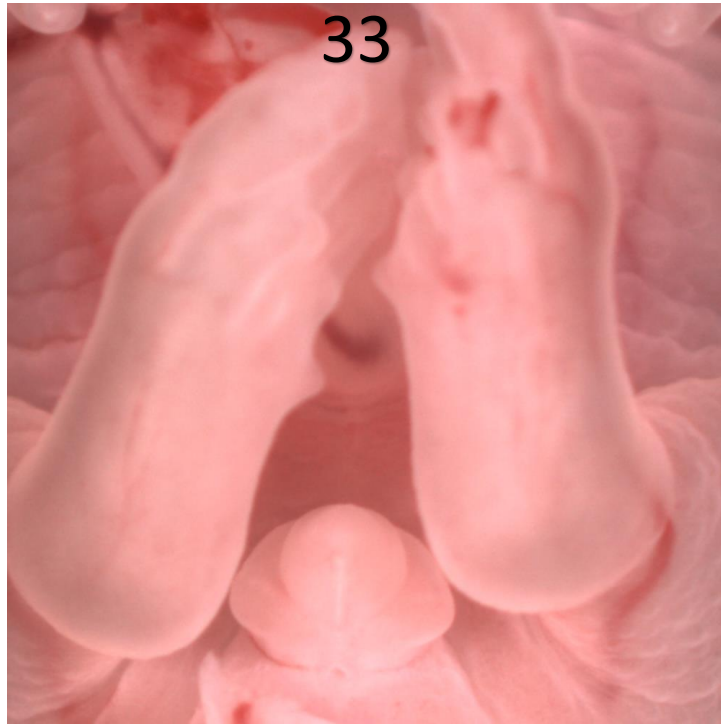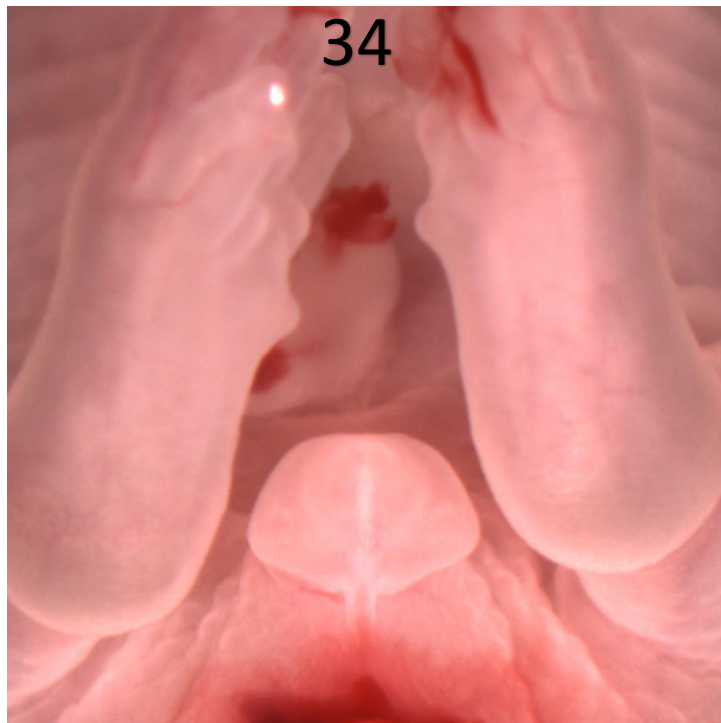

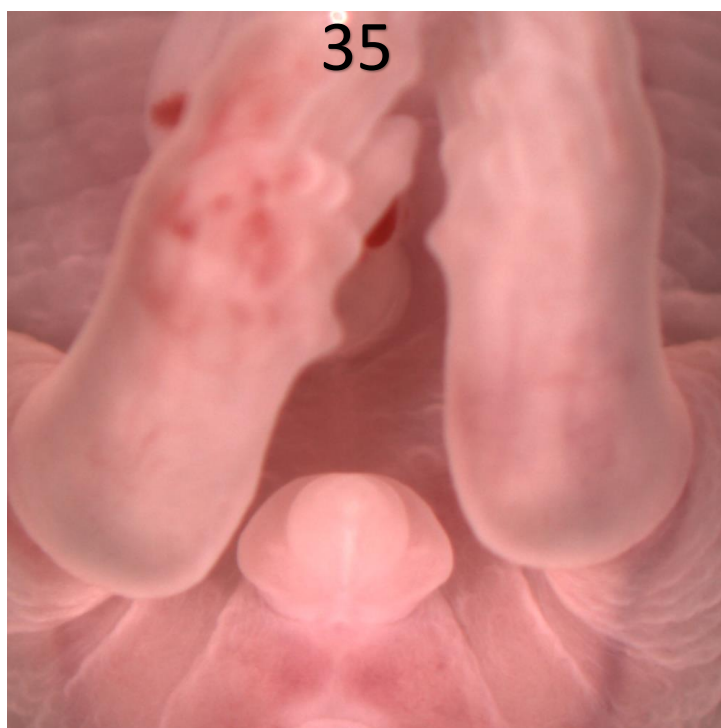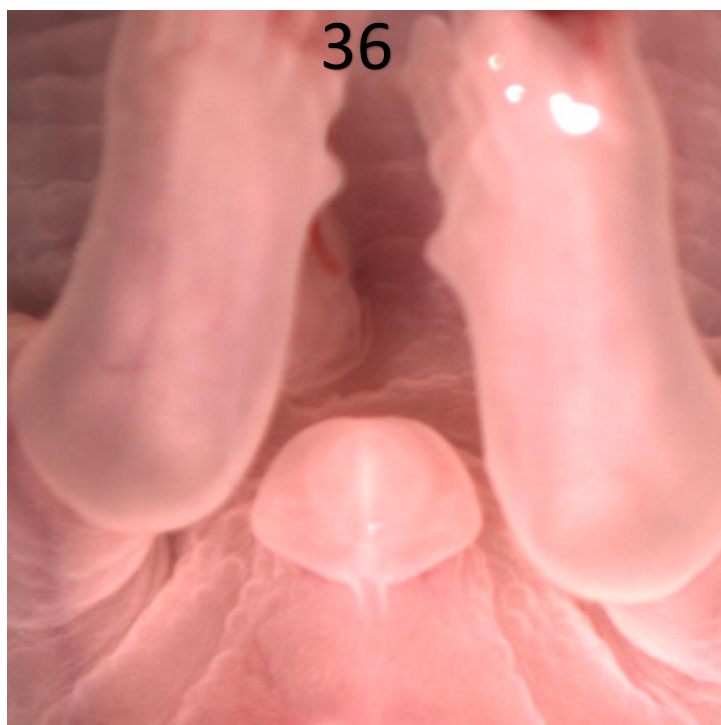

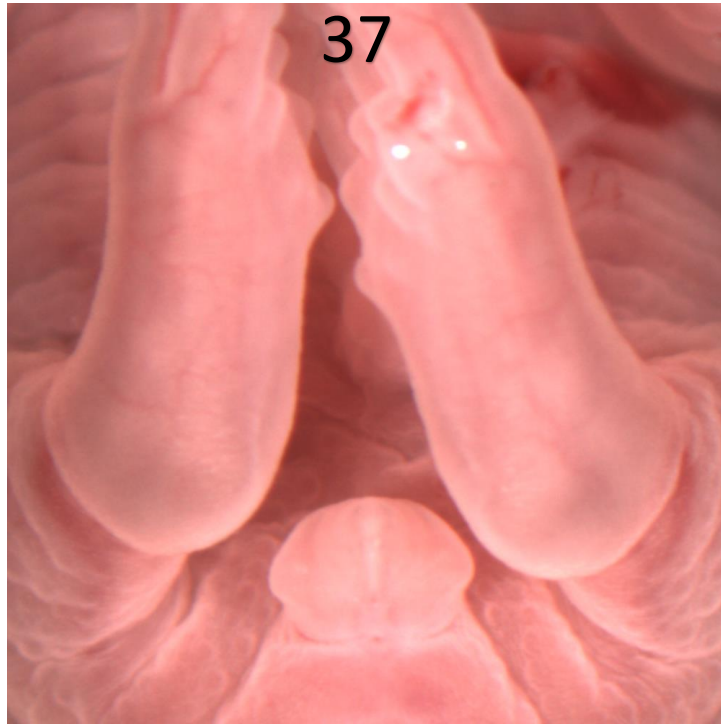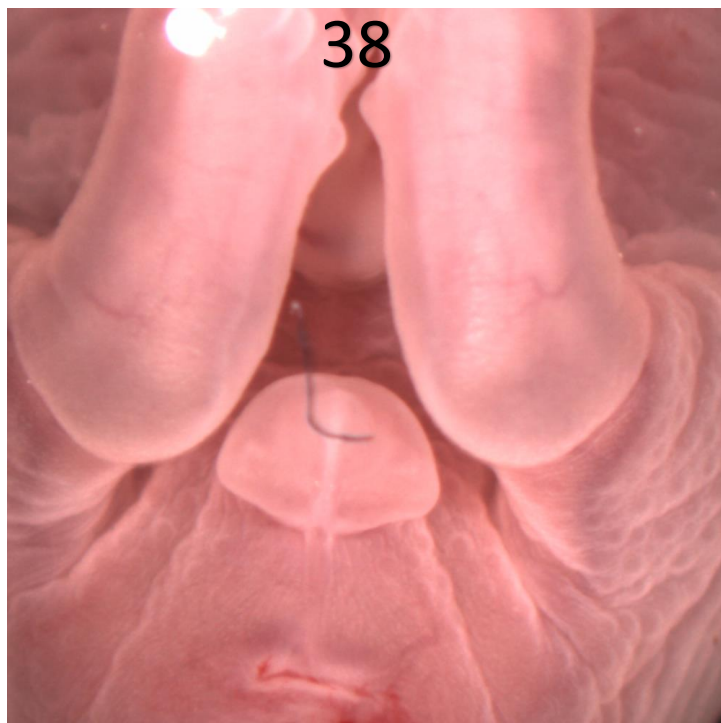

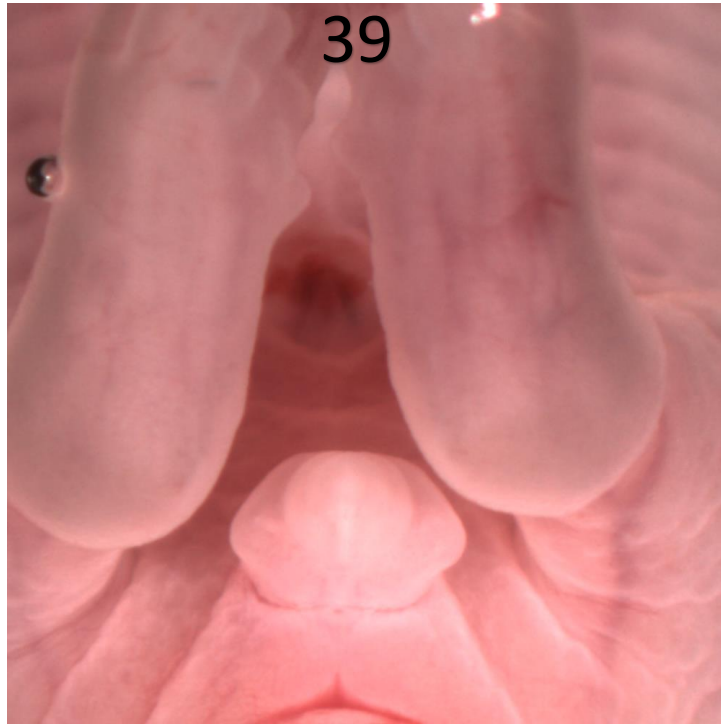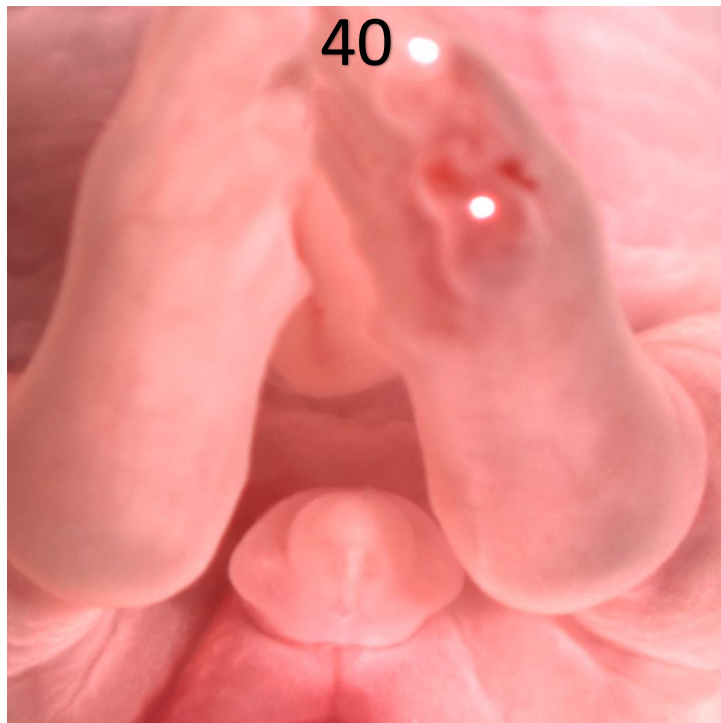

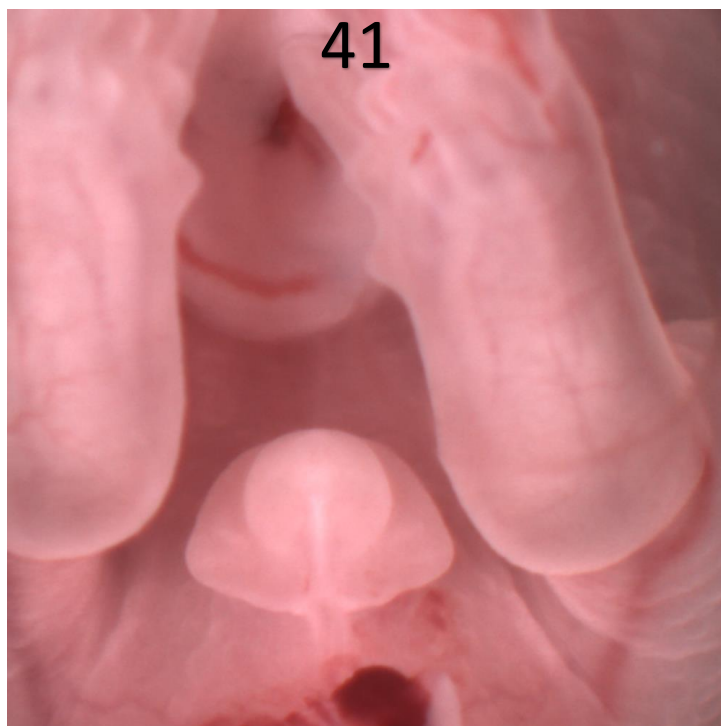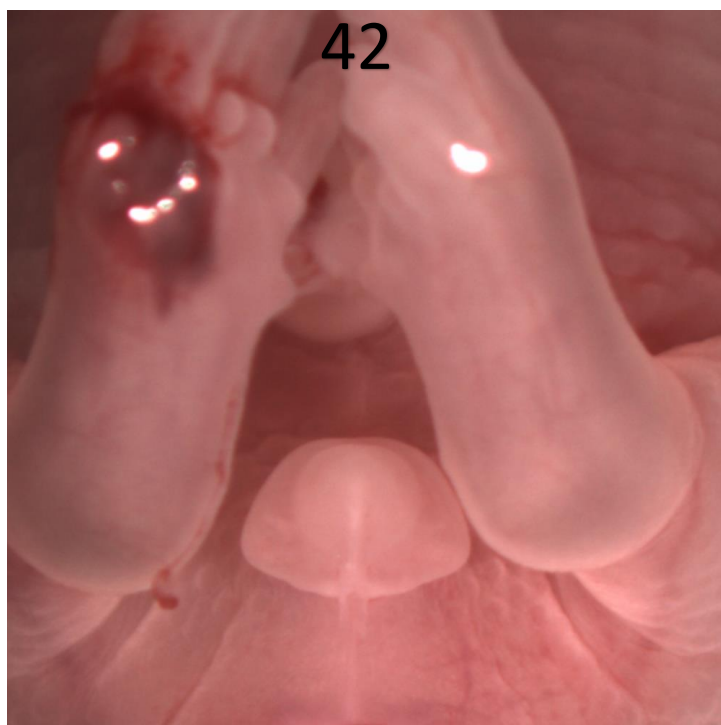

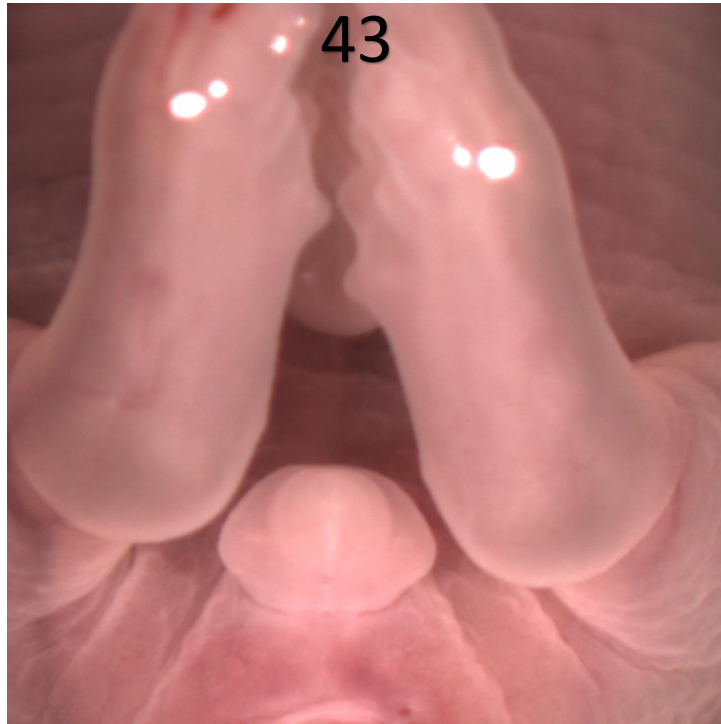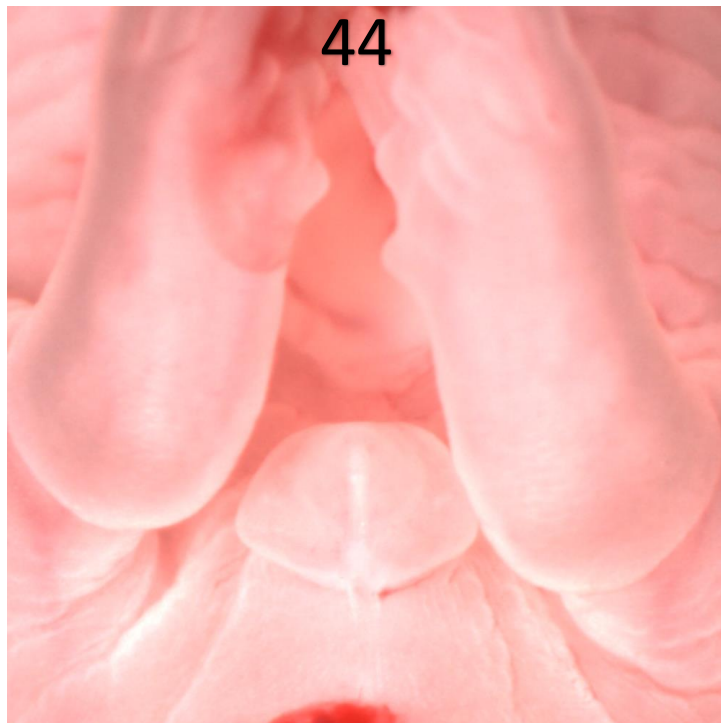

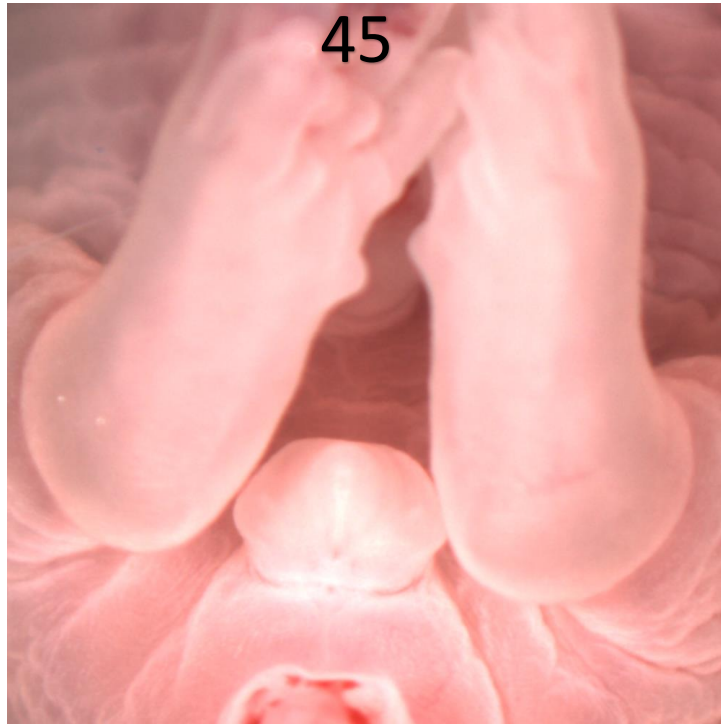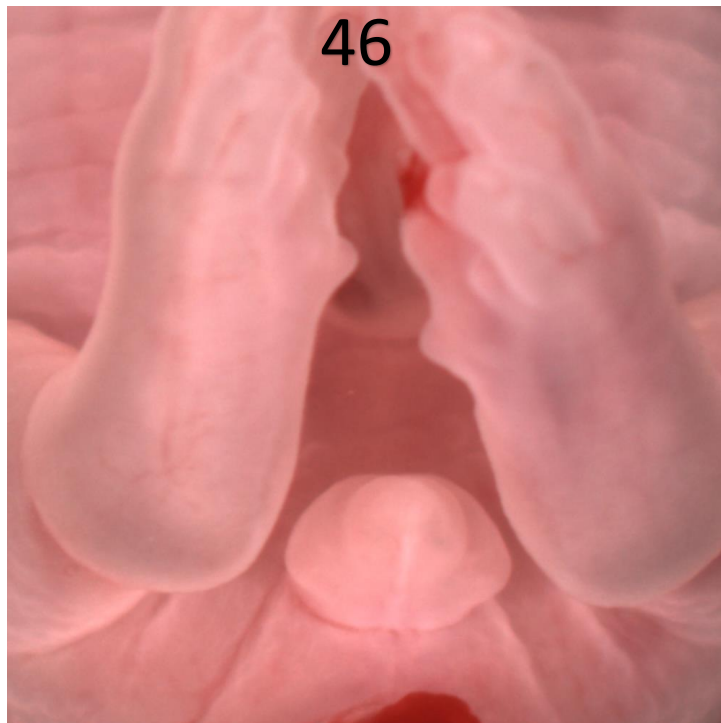

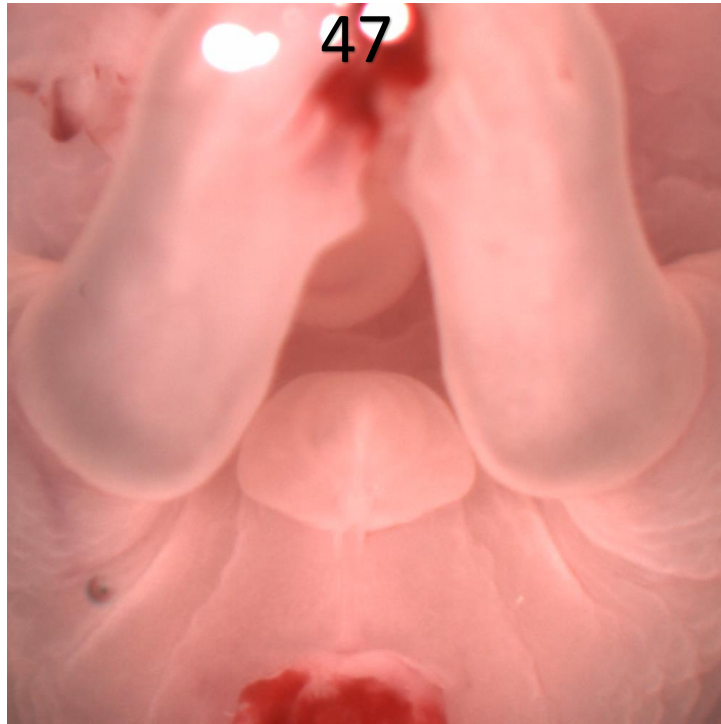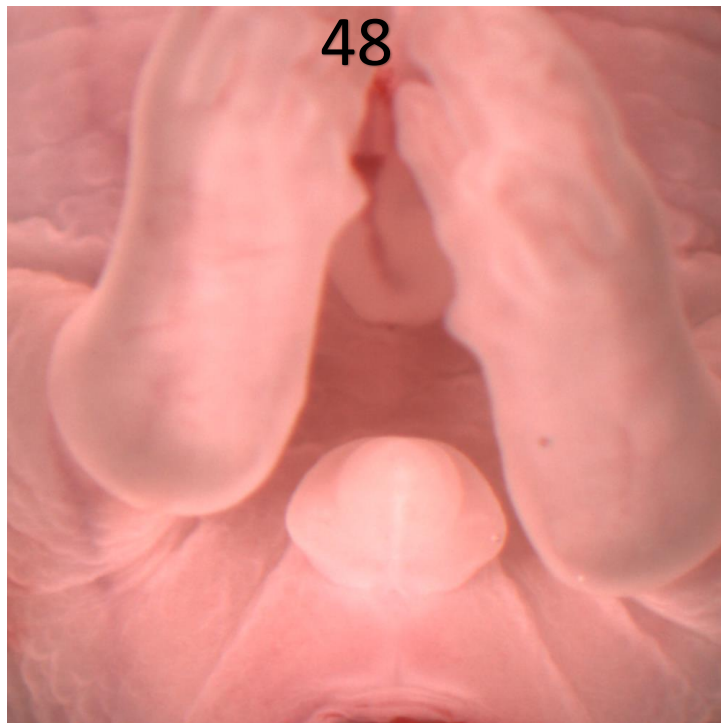

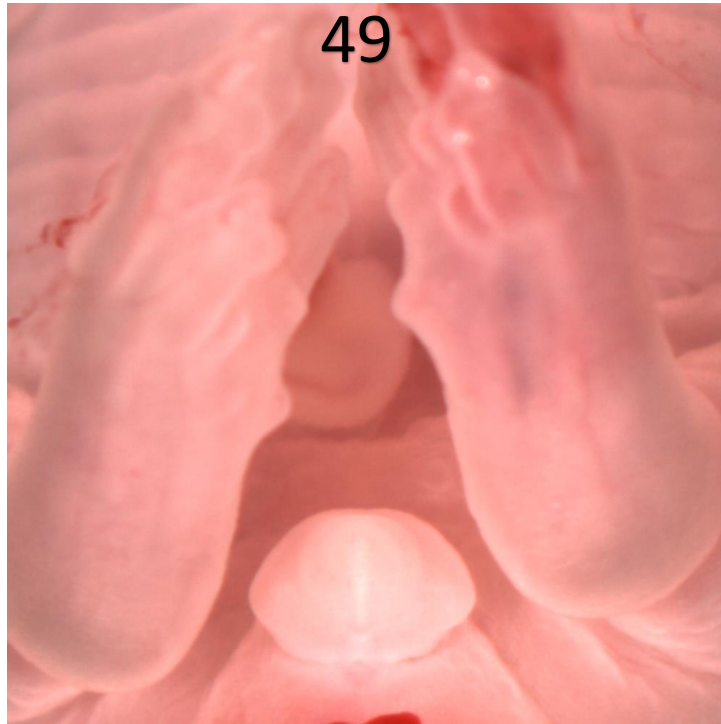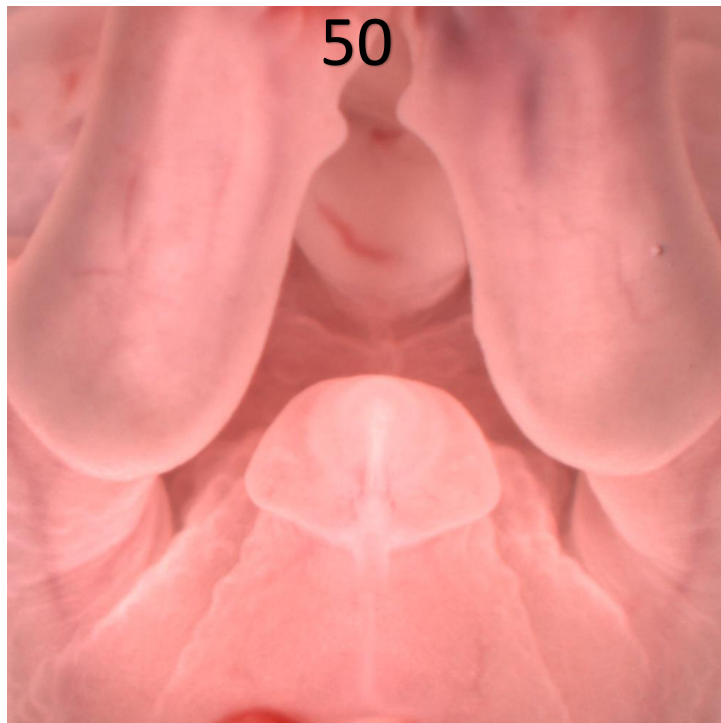

## Answers

- |         |         |         |         |         |
|---------|---------|---------|---------|---------|
| • 1. F  | • 12. M | • 23. F | • 34. M | • 45. F |
| • 2. M  | • 13. F | • 24. F | • 35. F | • 46. F |
| • 3. M  | • 14. M | • 25. M | • 36. M | • 47. M |
| • 4. F  | • 15. M | • 26. F | • 37. F | • 48. F |
| • 5. F  | • 16. F | • 27. M | • 38. M | • 49. F |
| • 6. F  | • 17. F | • 28. M | • 39. F | • 50. M |
| • 7. M  | • 18. M | • 29. M | • 40. F |         |
| • 8. F  | • 19. F | • 30. F | • 41. M |         |
| • 9. F  | • 20. M | • 31. M | • 42. M |         |
| • 10. M | • 21. M | • 32. F | • 43. F |         |
| • 11. M | • 22. M | • 33. F | • 44. M |         |

Supplement: S1 File — Set of 50 numbered photographs of genitalia on GD 17.0, and key to their sex. Photos are near optimal, with little to no instances of feet or blood obscuring any features, making them ideal for training. (PDF) [file pone.0194767.s004.pdf]
